# Practical Bayesian Inference in Neuroscience: Or How I Learned to Stop Worrying and Embrace the Distribution

**DOI:** 10.1523/ENEURO.0484-23.2024

**Published:** 2024-07-19

**Authors:** Brandon S. Coventry, Edward L. Bartlett

**Affiliations:** ^1^Department of Neurological Surgery and the Wisconsin Institute for Translational Neuroengineering, University of Wisconsin-Madison, Madison, Wisconsin 53705; ^2^Weldon School of Biomedical Engineering, Department of Biological Sciences, and the Institute for Integrative Neuroscience, Purdue University, West Lafayette, Indiana 47907

**Keywords:** auditory, Bayesian inference, neural coding, neural data analysis, statistical inference

## Abstract

Typical statistical practices in the biological sciences have been increasingly called into question due to difficulties in the replication of an increasing number of studies, many of which are confounded by the relative difficulty of null significance hypothesis testing designs and interpretation of *p*-values. Bayesian inference, representing a fundamentally different approach to hypothesis testing, is receiving renewed interest as a potential alternative or complement to traditional null significance hypothesis testing due to its ease of interpretation and explicit declarations of prior assumptions. Bayesian models are more mathematically complex than equivalent frequentist approaches, which have historically limited applications to simplified analysis cases. However, the advent of probability distribution sampling tools with exponential increases in computational power now allows for quick and robust inference under any distribution of data. Here we present a practical tutorial on the use of Bayesian inference in the context of neuroscientific studies in both rat electrophysiological and computational modeling data. We first start with an intuitive discussion of Bayes' rule and inference followed by the formulation of Bayesian-based regression and ANOVA models using data from a variety of neuroscientific studies. We show how Bayesian inference leads to easily interpretable analysis of data while providing an open-source toolbox to facilitate the use of Bayesian tools.

## Significance Statement

Bayesian inference has received renewed interest as an alternative to null significance hypothesis testing for its interpretability, ability to incorporate prior knowledge into current inference, and robust model comparison paradigms. Despite this renewed interest, implementations of Bayesian inference are often stymied by undue mathematical complexity and misunderstandings underlying the Bayesian inference process. In this article, we aim to empower neuroscientists to adopt Bayesian statistical inference by providing a practical methodological walkthrough using single and multiunit recordings from the rodent auditory circuit accompanied by a well-documented and user-friendly toolkit containing regression and ANOVA statistical models commonly encountered in neuroscience.

## Introduction

The scientific process generates new experiments and updates models based on quantitative differences among experimental variables, evaluated by statistical inference. Inference tools are foundational to these studies, providing the necessary machinery to make decisions and conclusions from data. Frequentist-based null significance hypothesis testing (NSHT) has been the gold standard of inference in neuroscience and science at large in part due to the computational simplicity of frequentist models compared with permutation sampling or Bayesian-based methods. A significant problem present in the current practice of NSHT, however, arises in the adoption of the *p*-value as the de facto metric of experimental “success,” notorious for its difficulty in interpretation and correct usage (Krueger and Heck, 2019). The confluence of exponential increases in computational power with the wider discussion of problems with NSHT usage has created renewed interest in Bayesian inference as an alternative to frequentist NSHT while offering interpretability benefits over the *p*-value and NSHT overall.

The use of *p*-value thresholds as an ubiquitous decision rule in frequentist methods is fraught with problems due to fundamental misunderstandings of its use, its interpretability, and most pathologically, its susceptibility to intentional and unintentional *p*-hacking (Nuzzo, 2014). Contrary to the initial intent of Ronald Fisher (Fisher, 1992), the *p*-value has often become the gatekeeper of significance in studies. In this role, it limits deeper observations into data, and it is often used without proper experimental design to ensure proper use and control. Statistical inference methods require first defining a statistical model with the power to adequately describe the data-generating process. Inference is then performed to estimate the population distribution from limited samples of observed data. Once estimates of population distributions are made, the determination of whether or not these distributions represent a significant effect is determined. NSHT is somewhat a victim of its own success, where common practice has distilled the practice of NSHT to chase the somewhat arbitrary *p* < 0.05 measure of significance devoid of model or data considerations (Krueger and Heck, 2019). Furthermore, even in the best of experimental designs, the *p*-value is a surrogate for arguably what a researcher is most interested in: how likely is it that observed data have some effect different from null (Kruschke, 2011; Gelman and Shalizi, 2013).

Bayesian methods offer a solution to the problem of pathological *p*-value use and interpretation, providing a direct measure of the probability that observations have some significant effect (Kruschke, 2011; Gelman and Shalizi, 2013). This is done by reallocation of the probability of possibilities as parameters in a mathematical model of the data-generating process, leading to probabilistic estimates desired by but not attainable with *p*-value analyses. Bayesian methods are inherently data driven; models are built with prior knowledge directly incorporated from parameters estimated directly from observed data.

Bayesian inference, though developed earlier than frequentist approaches, was not adopted as the primary inference paradigm due to the computational demands necessary to solve inference problems outside of certain canonical forms (Bishop, 2006) and the later adoption of the frequentist interpretation of probability (Fienberg, 2006). Inference on arbitrary distributions required a deeper mathematical knowledge and computation of integrals that were potentially intractable without modern numerical integration techniques. Frequentist paradigms however were more easily adapted to computationally simple algorithms, allowing researchers to “do statistics” without extensive formal training. However, exponential increases in computational power with the development of powerful Markov chain Monte Carlo (MCMC) sampling methods now allow researchers to perform meaningful Bayesian inference on arbitrary distributions underlying observed data (Gilks et al., 1996). While Bayesian approaches have received some attention for inference in electrophysiology (Wood et al., 2004; Wu et al., 2006; Vogelstein et al., 2009; Cronin et al., 2010; Gerwinn et al., 2010; Park and Pillow, 2011), the advantageous interpretability of inference and data-driven nature found in Bayesian statistics have as of yet been widely used in neuroscientific studies. This is in part a pedagogical problem, in that most neuroscientists do not encounter Bayesian statistics during formal training combined with the perception that a high level of mathematical acuity is necessary to perform Bayesian inference.

The goal of this tutorial is to remedy the opacity that often accompanies discussions of Bayesian inference by providing simple, step-by-step walkthroughs of Bayesian inference with four common inference paradigms utilizing tools that facilitate Bayesian computation for users at all levels of mathematical skill. We also aim to demonstrate the explanatory power of Bayesian inference in the context of neuroscience data. While the aim of this article is focused on application, this tutorial will begin with a brief introduction to Bayes' rule and its constituent components necessary for inference. For more theoretical and mathematical considerations of Bayesian inference, see the following books and articles: Gerwinn et al. (2010), Colombo and Seriès (2012), Bielza and Larranaga (2014), Kruschke (2014), Kruschke and Vanpaemel (2015), Ma (2019), Gelman et al. (2021), Van De Schoot et al. (2021), and Cinotti and Humphries (2022).

### Outline of Bayesian methods

To best facilitate practical application of Bayesian inference methods in neuroscience, a variety of datasets were acquired and analyzed. Data acquisition paradigms are described below in the Materials and Methods section. Bayesian inference is introduced in the context of regression to infer changes in inferior colliculus (IC) single-unit firing from changing auditory stimuli. *T* test-like group comparisons are demonstrated using computational models of basal ganglia–thalamocortical (BGTC) local field potentials (LFPs). Bayesian implementations of repeated measures and random effects models are demonstrated using chronic, multichannel single-unit recordings from the auditory cortex. Finally, Bayesian ANOVAs and ANCOVAs are utilized in assessing age-related changes in IC single-unit firing. All model implementations are available in our Bayesian inference for neuroscience toolbox at https://github.com/bscoventry/Practical-Bayesian-Inference-in-Neuroscience-Or-How-I-Learned-To-Stop-Worrying-and-Embrace-the-Dist and permanently indexed in our Zenodo repository at 10.5281/zenodo.11206116.

### A simple example of Bayesian inference in neuroscience

**Figure 1.**
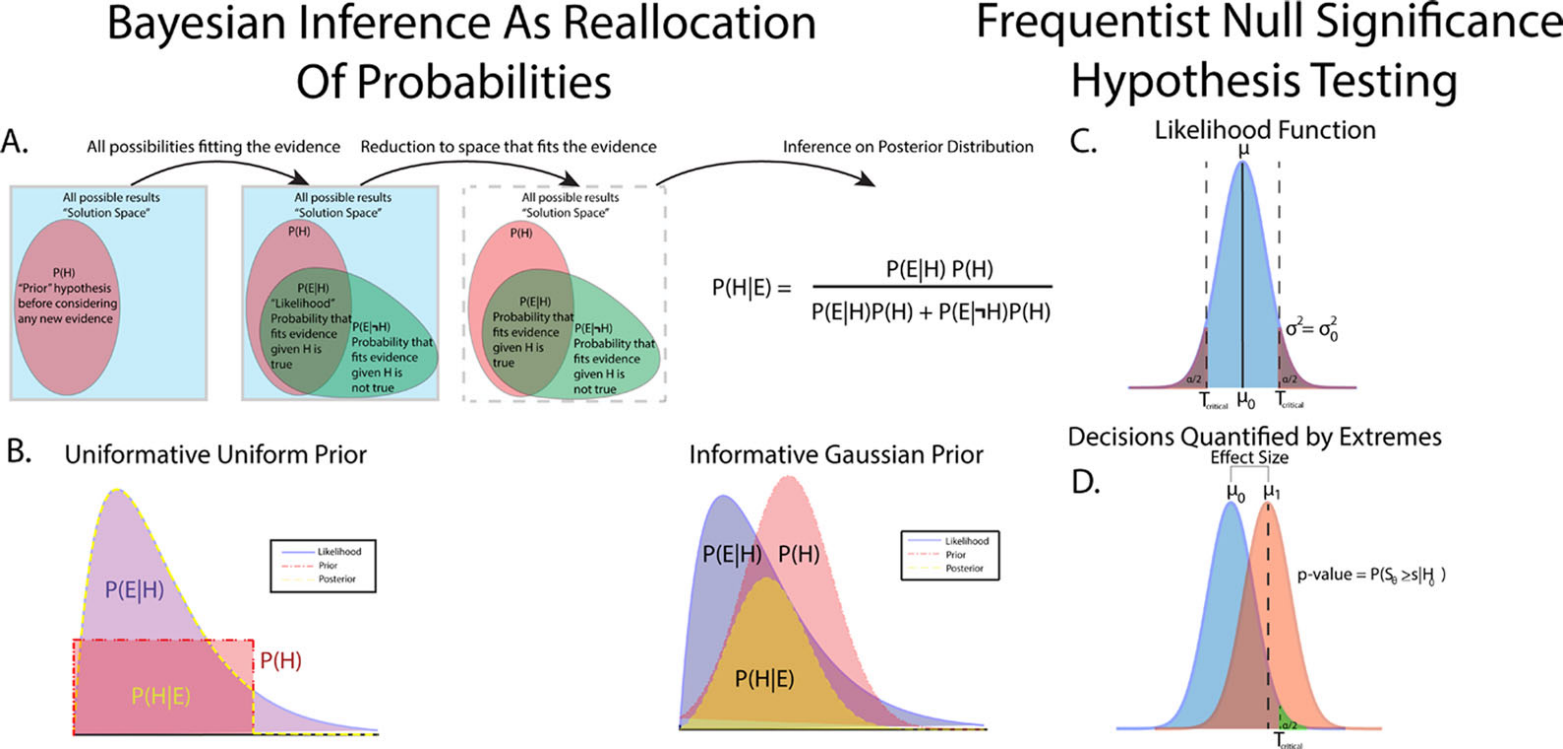
Graphical description of Bayes’ rule and the interaction between prior distributions and likelihood functions leading to the final posterior distribution. ***A***, Bayes’ rule can be thought of as a reallocation of probability to the posterior after accounting for prior distributions and observed evidence. ***B***, An example of a posterior generated from an inverse-gamma-distributed likelihood and a uniformly distributed prior. Uniform priors reflect the likelihood function, and thus the observed data with no redistribution of probability, making uniform distributions uninformative priors. However, care must be taken in using uniform distributions as observed data outside of prior bounds are mapped to zero probability. A second example of a posterior generated from an inverse-gamma-distributed likelihood and a Gaussian-distributed prior shows how a prior can be considered informative (right). In this posterior, the prior “shapes” the posterior to a greater extent than a uniform prior. Well-designed priors in neuroscientific data can thus shape posteriors away from responses that are not physiological and can be vetted in the statistical decision, data review, and postpublication review stages. Prior distributions with longer tails can handle extremes of observed data by mapping extreme events to low, but nonzero, representation in the posterior. Example B represents extremes of prior choices, with minimally informative priors often chosen to let the data “speak for itself” with little change to posterior from prior influence. ***C***, Comparison of Bayesian and frequentist statistics. NSHT utilizes a parameterized likelihood function that describes the data-generating process. ***D***, Decisions in NSHT are generally made based on quantification of the probability of observing test results as extreme as observed data assuming that the null hypothesis is true.

**Figure 2.**
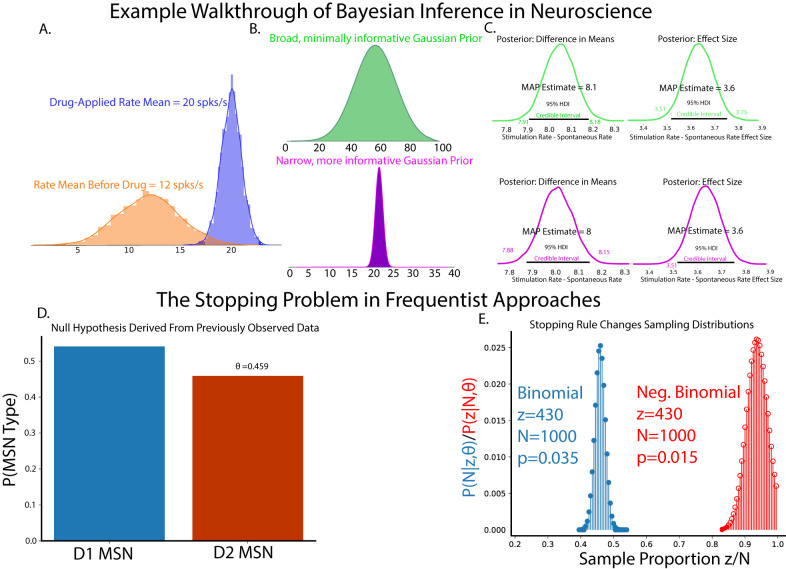
An illustrative example of how Bayesian inference might be used in an electrophysiological experiment. Prior distributions can be iteratively formulated by previous experiments drawn from both internal and published data. ***A***, Observation of data distributions shows that both outcomes follow normal distributions, guiding the choice of a normal likelihood distribution. ***B***, Examples of a minimally informative prior distribution (top) and a more informative prior distribution (bottom). Design of the prior distribution is guided by both expert knowledge by the investigators along side established published data. ***C***, Incorporation of more informative prior distributions results in a similar decision rule as the less informative prior, but with better estimation of credible model parameters. ***D***, The null hypothesis of a second hypothetical example demonstrating *p*-value sensitivity to stopping rules. ***E***, Given the same data and sample size, stopping rules can fundamentally alter the sample distribution estimate and give conflicting *p*-value results.

As a simple, motivating example of how Bayesian statistics can be performed in neuroscientific studies, consider a hypothetical in vitro whole-cell patch-clamp experiment recording of a cortical neuron (Figs. 1, 2*A–C*). This example will serve as a concrete example for explaining the components used in Bayesian inference. The experimental details are less important than the formulation of Bayesian models, but are given in some detail to help visualize the hypothetical study. Consider a study to assess the effect of a novel drug on firing rates in L2/3 cortical pyramidal neurons. The goal of the study is to assess the change in the firing frequency versus injection current before and after drug application during current-clamp recording. To complete Bayesian inference, the state of knowledge of firing rates should be known and quantified in a structure known as the prior distribution, and the observed data are constructed into a structure called the likelihood function. The prior distribution and likelihood function are then used to create a posterior distribution from which all statistical inference is derived.

### An introduction to Bayesian inference

Bayes' rule forms the basis for all Bayesian inference as the machinery to reallocate probability from prior distributions and observed data to the posterior. While a full mathematical and computational treatment of Bayes' rule is out of scope for this article, we begin by outlining the components of Bayes' rule, how inference is performed, and show, using the above hypothetical experiment, how priors and likelihood functions can be formed.

### Bayes' rule

Foundational to Bayesian approaches is a complementary, but epistemically differing view of probability from that of frequentist approaches. While the frequentist perspective treats probability as the relative frequency of the occurrence of some event, the Bayesian perspective instead treats probability as the expectation of an event occurring which can be used to quantify not only the state of knowledge of an event but also the uncertainty involved in measuring an event (Bishop, 2006; Kruschke, 2011, 2014; Van De Schoot et al., 2021). Traditionally, the Bayesian perspective has been called “belief” (Kruschke, 2010), a perhaps unfortunate name that belies the fact that the Bayesian perspective of uncertainty of an event is fundamentally quantifiable. Perhaps a better description of Bayesian belief is instead quantification of the state of knowledge by accounting for uncertainty. The cornerstone of Bayesian inference is Bayes’ rule, defined as follows:

$$P(H|E)=\;\frac{P(E|H)P(H)}{P(E)},$$

where *H* is the quantification of the state of a hypothesis and *E* is the quantification of observed evidence. In the context of inference, it is helpful to explicitly state the role of the model in Bayesian formulations:

$$P(\theta |E,M)=\frac{P(E|\theta ,M)P(\theta |M)}{P(E|M)},$$

where *M* is the model of the data-generating process and θ are the model parameters. The individual components of Bayes' rule are given names corresponding to the purpose they serve, with P(θ|E,M) called the posterior distribution, P(E|θ,M) the likelihood function, P(θ|M) the prior distribution, and P(E|M) the evidence or marginal likelihood function. Taken together, Bayes' equation represents the quantification of observed data accounting for prior knowledge (Fig. 1*A*). Each component plays a key role in Bayesian inference, and each will be discussed briefly below.

### The model evidence

The denominator term P(E|M), called the model evidence (or just the evidence or marginal likelihood in Bayesian parlance), is the quantification of the probability of observing the data under a chosen model of the data-generating process. At first glance, the calculation of the total evidence appears to be an insurmountable task. In reality, this term is the weighted average of parameter values in a given model weighted by the relative probability of a given parameter value (Kruschke, 2014) and thus acts as a normalization term to ensure the numerator is a proper probability distribution. The structure of P(E|M) will change based on whether the distributions represent probability mass functions (discrete case) or probability density functions (pdfs; continuous case). In the discrete case, the evidence is as follows:

$$P(E|M)=\underset{\theta }{\sum }\;p(E|\theta ,M)p(\theta |M),$$

and in the continuous case as follows:

P(E|M)=∫p(E|θ,M)p(θ|M)dθ.

The evidence function thus represents an average of the likelihood function across all parameter values conditioned on the prior distribution. The marginal likelihood can also be utilized to assess the plausibility of two competing models (Johnson et al., 2023). The evidence, especially in the continuous case, is historically what made Bayesian inference difficult due to the need to evaluate a complex integral numerically. However, the advent of MCMC methods with improvements in personal computer processing power has allowed for computationally efficient integration without the need for supercomputing hardware. MCMC methods will be discussed in a subsequent section.

### The prior

The prior, P(θ|M), is often the major stumbling block for those entering into Bayesian inference, but this hurdle is less about the prior, and more about what the prior is perceived as. The prior, P(θ|M), describes the investigators’ prior beliefs on the state of knowledge of the study. While normally distributed priors are common, the prior can take the form of any proper probability distribution. Critics of Bayesian inference have described the prior as purely subjective, but we, and many others (Kruschke, 2010; Box and Tiao, 2011; Gelman and Shalizi, 2013), argue that the prior represents an explicit declaration of the investigators’ knowledge, assumptions, and the general state of a field which is implicit and often is present but not stated in frequentist approaches. Moreover, one is encouraged to perform prior predictive checks to compare the sensitivity of competing priors in a Bayesian inference model, as we will show subsequently. The practice of the design of experiments and their resulting publications are rife with implicit priors that are often not acknowledged or realized when reporting results. As an example, consider the study of cortical extracellular single-unit recordings (Paninski et al., 2004; Bartlett and Wang, 2007; De La Rocha et al., 2007; Coventry et al., 2024). The investigator could be leading a project with vast knowledge accumulated over years of study. Or the investigator is a trainee of a career researcher who draws a view of cortical physiology from their experienced mentor mixed with reading current literature. When designing an experiment, the investigator will have some intuition regarding likely and biologically feasible resting state and stimulus-evoked firing rates, cognitively assigning a relatively low likelihood to seeing extremes of firing rates with higher likelihood assigned to moderate firing rates previously observed in literature or seen in experiments, and likely will discard or treat as outliers extreme firing rates or artifacts thought to be nonbiological noise. The power of the prior distribution in Bayesian approaches is in part the need to explicitly quantify and report these prior beliefs, which can be analyzed and scrutinized as part of the peer review or postpublication process. Prior distributions also require investigators to consider their biases and relative expectation on the importance of previously recorded and read data, promoting a deeper understanding of not only the data obtained within their lab but also of the general state of the specific neuroscience field. As the name implies, prior beliefs are quantified as probability distributions by the investigators. In our hypothetical experiment on cortical neurons, the graduate student surveys the knowledge (i.e., a literature search) surrounding these neurons and assigns a broad, normally distributed prior (Fig. 2*B*, top) centered around the average firing rates of these cortical neurons across all studies surveyed before the experiment is conducted. The logic to this prior is that while firing rate distribution to this novel drug is not known, the totality of observed firing rates reasonably approximates the range of possible firing states of that neuron.

This begs the question as to what a prior might look like in newer avenues of study where a paucity of data exists or in situations where researchers and data analysts want the data to “speak for itself” outside any influence of the prior. In these cases, priors can be designed to be “noninformative” or “weakly informative,” assigning broad, noncommittal distributions to the prior. One might assign a uniform distribution on the prior, effectively treating each parameter outcome as equally likely. Uniformly distributed priors do require some caution, however, as any parameter value outside of the bounds of the uniform distribution is automatically assigned probability 0 in the posterior, even if that value has been observed (Fig. 1*B*, left). In many cases, it is better to allow small, but nonzero, probabilities to extreme values, such as the tails of a normal distribution, such that evidence for unexpected events is represented in the posterior given strong data (Fig. 1*B*, right). Conversely, priors can be made to be highly informative in situations where physiological bounds are well known and well studied, where extreme values are known to be biophysically irrelevant, impossible, or known to be due to instrument noise (e.g., large 50/60 Hz noise peak in power spectrum indicative of wall power noise).

### The likelihood

The likelihood function, P(E|θ,M), describes the probability that data is observed given parameter values θ in a data-generating model *M*. In the context of inference, the likelihood function updates information given in a prior distribution to the posterior distribution given the observed data (Etz, 2018). The likelihood function is generally not a proper distribution, in that it is conditioned on yet unknown parameters and may not integrate to 1, but the evidence and prior terms ensure that the resultant posterior distributions are true probability densities. The idea of the likelihood function is present in both Bayesian and frequentist models, but has vastly different interpretations. The model parameters in a frequentist viewpoint converge upon singular values learned, usually though maximum likelihood estimation, from merging competing hypotheses of data. Bayesian approaches treat model parameters as ranges arising from distributions after observing the data at hand. For our hypothetical cortical slice experiment, the graduate student runs the study, plots the distribution of observed results, and finds that drug application produces small increases in mean firing rates that are very consistent between trials and cells, as evidenced by relatively tight distributions of data (Fig. 2*A*). However, the overlap of naive and drug conditions means that quantification of the uncertainty of measurement of the drug effect must be performed. The distributions of evoked firing rates as a function of injected currents are the data encoded in the likelihood function. Upon observation of the acquired data, both the naive and drug applied groups appear to be generated from a normally distributed underlying process. As such, the choice of a normally distributed likelihood function conditioned on the observed data is a good choice. This choice will be validated after the posterior is derived using a process called posterior predictive checking which is described below.

### The posterior

The prior, likelihood, and evidence then form the posterior P(θ|E,M), the reallocation or mapping of probability from likelihood function, prior, and model evidence to an all-encompassing distribution. The posterior thus is the evidence for parameters θ conditioned on observed data and a model of the data-generating function. The posterior forms the basis for inference, with all relevant information encoded in its distribution. Inference on the posterior distribution is covered in a section below.

### Estimation of the posterior

Historically, limited computational capacities posed a significant obstacle to the widespread adoption of Bayesian inference methodologies. Application of Bayes' rule requires computation of complex integrals, limiting early Bayesian inference to canonical prior to posterior transformations, called conjugate pairs. However, modern MCMC tools simplify the computation of these complex integrals, allowing for quick and easy estimation of arbitrary posterior distributions. MCMC involves the generation of random samples that converge to a target probability distribution, the details of which can be learned from the following reviews (Hoffman and Gelman, 2011; Betancourt, 2017).

### Making decisions on the posterior

We define inference broadly as the process by which reasoning and decisions about phenomena are made from a sample of observations of the phenomena. Incorporation of prior knowledge in Bayesian inference allows for optimal decision-making on observed data (Blackwell and Ramamoorthi, 1982). The posterior contains all necessary information to make inferences on experimental data incorporating prior knowledge. However, it is best to consider the specific goals of inference before performing statistics. Possible goals of inference are as follows (Kutner et al., 2005; Kruschke, 2014):

- Infer the parameters of a model.
- Reject or confirm a hypothesis, null or otherwise.
- Compare two or more competing models.

In the case of neuroscientific studies of unit electrophysiology, inferring model parameters occurs when an experiment aims to establish how neural firing rates change with changes in applied stimuli. Or one may want to confirm or reject a null hypothesis that a treatment has the desired effect or that there are differences between neural populations. Importantly, because the Bayesian inference operates solely on the posterior distribution, one can confirm or reject competing hypotheses and not simply reject the null as in frequentist NSHT.

Regardless of the goal, inference always involves analyzing the posterior, which provides a complete representation of the distribution of a given parameter given the experimental data. Therefore, decisions about the data, the effect of model parameters, and/or which hypothesis has more evidence are performed with calculations on the posterior. There are multiple decision rules that can be used to assess the posterior. The most common, and in our opinion, the most intuitive is that of the Bayesian credible interval. The credible interval is a statement of probability, describing the probability of a parameter estimate of falling within a given range. As credible intervals are not strictly unique, Bayesian inference convention is to fix the interval to the smallest interval which contains 95% of the posterior distribution density mass called the highest density interval (HDI). Observations of posterior HDIs can then be used to assess the relative effect of a parameter. Regions of practical equivalence (ROPEs) may be included in the posterior distribution that explicitly define a range of values that are effectively equivalent to a null value, with parameters considered significant if 95% of the posterior distribution (95% HDI) does not contain 0 or any values in the ROPE (Kruschke, 2018). Along with posterior HDIs, calculations of maximum a posteriori (MAP, distribution mode) estimates from the posterior are performed to quantify a most likely parameter value. While decision rules are important to assess the relative effect of statistical model parameters, we reiterate that simply passing a decision rule should not conclude the inference step. Inference should be made in the context of the evidence presented in model quality checks, observed data posterior distributions, and decision metrics. Returning to our example of cortical neuron firing rates, the graduate student takes the derived prior formed by survey of previous literature and the likelihood formed by observed data and uses Bayes' rule to calculate posterior distributions. Plots of the posterior distributions and inference from the posterior show that application of the drug significantly increases cortical neuron firing rates (Fig. 2*C*, top; MAP estimate = 8.1). This effect is considered significant because the 95% HDI does not contain 0. This means that, given the observed data, the chance that there is no difference between groups has an upper bound of 5%. Given that the value 0 is far within the tail of the posterior distribution, the chance of no difference between groups is vanishingly small (but easily quantifiable if necessary). Measurement of the effect size distribution shows that the observed effect is most likely 3.6, with the probability of no effect also being vanishingly small. After completion of the experiment, the advisor of the graduate student noticed that both in vitro and in vivo data were included in the prior, even though firing rates differ dramatically between those preparations in the previous work. Rerunning the analysis with the highly informed prior (Fig. 2*B*, bottom) based only on previously observed in vitro data produces a similar result, but with a slight reduction in the uncertainty in the difference of group means as measured by group mean difference MAP estimates and 95% HDIs. In this case, comparing weakly and strongly informed models also shows that relatively drastic changes in the prior make minor changes in posterior estimates (Fig. 2*C*, bottom), suggesting that observed data are the primary drivers of inference and are not unduly influenced by the prior. Taken together, both broad, minimally informative and highly informative priors often lead to similar decisions on the data and are useful for cases where prior knowledge is sparse. However, when prior knowledge is available, incorporating more information in the form of an informative prior backed by data can provide reduction in uncertainty in the estimation of study effects and better handle outliers within data.

This simple example shows one way in which priors and likelihoods are defined in neuroscience studies in a manner that most researchers unknowingly do by running experiments and reading the literature. The prior serves to take intrinsic prior knowledge and quantify it to provide more transparent reporting of experimental results. Furthermore, data forms the likelihood function, facilitating inference that makes decisions on the data at hand while accounting for prior knowledge of the state of a particular field. It also introduces the idea of using model comparison to assess prior influence on inference, which will be rigorously explored below.

### Inference using NSHT

While theoretical comparisons of Bayesian inference versus NSHT are out of scope for this article, a brief description of decision-making using NSHT is warranted to orient Bayesian inference. For detailed descriptions and discussions of NSHT, see the following reference texts and articles: Kutner et al. (2005), Nieuwenhuis et al. (2011), Maris (2012), Kruschke (2014), and Bzdok and Ioannidis (2019). Proper NSHT experimental design is performed before data acquisition with sample size, statistical tests, and significance levels set to achieve a desired level of statistical power. However, proper NSHT experimental design is largely not followed, in part due to lack of proper statistical training and external career pressure (Munafò et al., 2017) as well as the relative difficulty in data acquisition (Button et al., 2013). We believe that Bayesian approaches provide a way to do statistics more in line with current neuroscientific practice, in which beliefs about data are updated as data are acquired, informed by the current state of the field. Statistical power relies on estimates of population variances and differences in group means. We note that this is somewhat analogous to the declaration of prior distributions in Bayesian inference. After data are acquired using the predefined stopping rule, NSHT inference is then performed. Similar to Bayesian inference, NSHT utilizes a parameterized likelihood function (Fig. 1*C,D*) that describes the data-generating process. The null hypothesis is the likelihood function descriptive of a nonexistent effect. Statistical significance is most commonly assessed through the *p*-value metric, a measure quantifying the probability of obtaining a value of a given test statistic at least as extreme as the test statistic observed under the assumption of a null hypothesis. Another common metric for statistical significance and parameter estimation is the confidence interval, which utilizes sample distributions to estimate parameters of the population distribution. Importantly, unlike data-driven Bayesian approaches, both *p*-values and confidence intervals draw inference using in part data that are not observed, owing from calculations over hypothetical replications of an experiment (Wagenmakers, 2007; Kruschke, 2014). Comparisons between Bayesian methods and NSHT are summarized in Table 1.

**Table 1.** Brief comparison of Bayesian and frequentist inference paradigms

	Bayesian	Frequentist
Philosophy of probability	Quantification of uncertainty	Frequency of events
Data inference	P(hypothesis|data) “data driven”	P(data|hypothesis)
Decision rules	HDI, credible interval, ROPE, distribution of observed data distributions, Bayes’ factors	*P*-value, confidence interval, Type I/II error control
Model parameters	Distributions formed around uncertainty in observed data	Fixed but unknown values inferred from estimators (maximum likelihood/least squares)
Data distributions	Any distribution	Parametric models require data to follow normal distributions. Nonparametric methods are limited
Requires explicit declaration of prior distribution	Yes	No
Computational complexity	Higher	Lower
Advantages	Inference backed by evidence from observed data, a complete probabilistic description of experimental data, inference less dependent on sample size. Error control through prior and posterior predictive checks	Robust error control (with proper experimental design), quick and easy to implement

### Stopping problem bias in *p*-value calculation

A critical calculation in frequentist inference is the determination of the probability of correctly rejecting a null hypothesis when the alternative is true, called statistical power. As collecting data is an expensive venture, data collection stopping rules are defined such that a desired power is obtained while minimizing total samples needed to uncover a desired statistical effect. While this stopping rule is explicitly stated in preregistered studies designed to elucidate a particular effect, a large portion the neuroscientific literature does not state stopping rule intentions*.* It is, however, known that the choice of stopping rules fundamentally change *p*-value results (Lindley and Phillips, 1976; Berger and Berry, 1988; Frick, 1998). To illustrate this, consider another simple hypothetical neuroanatomical experiment [adapted from Kruschke (2014)]. The striatum is a critical juncture in the basal ganglia circuit of the motor pathway. Striatal function is governed by two classes of neurons differentially expressing D1 and D2 dopaminergic receptors, respectively (Parker et al., 2018). D1- and D2-expressing neurons are relatively homogeneously distributed in the dorsal striatum of the mouse, with the density of medium spiny neurons (MSNs) expressing D1 or D2 receptors found to be 108±5 and $95\pm 4\;10^{3}(\mathrm{cells}/\mathrm{mm})$, respectively (Gagnon et al., 2017). In the hypothetical example, researchers aim to validate whether this same distribution of D1- and D2-expressing MSNs is present in the rat striatum. After examining differentially labeled D1 and D2 neurons in immunolabeled slices of the rat striatum, it was found that out of a total of 1,000 neurons counted, 570 were labeled as D1-expressing MSNs and 430 were labeled as D2-expressing MSNs. Consider the case where the researcher stopped at an N=1,000 samples as it seemed that this sample size was large enough to determine an effect. In this scenario, the total number of samples is fixed, and the count of D2-labeled neurons is a random variable. Thus, MSNs are recorded as either D1 or D2 expressed as counts of binary decisions. Then, the probability of identifying a D2 neuron (*z*) from a total sample size of 1,000 (*N*) of MSN neurons is a binomial distribution:

$$p(z|N,\theta )=\left(\begin{array}{c} N\\ z \end{array}\right)\theta^{z}(1-\theta {)}^{N-z},$$

where θ is the probability of a given MSN neuron expressing D2 receptors. This model can also be expressed as the probability of a given MSN expressing D1 receptors with the model giving equivalent results. Given that the aim of the study is to assess whether rodent D1- and D2-expressing MSNs exist with the same distribution as mouse striatum MSNs, a null hypothesis of distribution of D2 to D1 neurons corresponds to the parameter θ=0.459 (Fig. 2*D*). In this scenario, we are considering whether counts are bigger or smaller than our null hypothesis, necessitating a one-tailed critical value of p<0.025. Distributions of this data using this stopping rule are shown in Figure 2*E*. Calculating the *p*-value for this data given this stopping condition results in p=0.035, failing to reject the null hypothesis.

Now, consider a second stopping rule. When asked why data collection was stopped, the researchers responded that identification of >525 D1-expressing MSNs and <500 D2-expressing MSNs was enough to establish the desired trends, with a total sample size equal to 1,000 occurring by chance. Note that while the data and sample size remain exactly the same, we no longer are discussing the probability of getting *z* counts in *N* samples; we instead have the probability of taking *N* samples to get *z* counts. This flips *N* from a fixed to a random variable and *z* from a random to a fixed variable. In this case, the probability of getting *z* counts in *N* samples is a negative binomial distribution (Fig. 2*E*):

$$p(N|z,\theta )=\frac{z}{N}\left(\begin{array}{c} N\\ z \end{array}\right)\theta^{z}(1-\theta {)}^{N-z}.$$

Calculating the *p*-value for this stopping rule results in a value of p=0.015, which does reject the null hypothesis. Notice that for the exact same data and sample size, the choice of stopping rule can fundamentally change the sampling distribution. This example serves as a cautionary tale in performing frequentist analyses *post hoc* without proper statistical design and consideration.

Bayesian approaches do not suffer from the same stopping problem rule. Being a “data-driven” approach, all data observations are codified in the likelihood function, which is independent of any stopping rule (Anscombe, 1954; Kruschke, 2010; Van De Schoot et al., 2021). Examples of the unchanging property of the likelihood function can be found in numerous studies (Berger and Berry, 1988; Gelman et al., 2009; Kruschke, 2014). Bayesian models can certainly be influenced in a similar way by choosing a prior distribution that does not appropriately reflect the state of knowledge of the data-generating process. However, as the prior distribution is reported explicitly and encapsulates the researcher's knowledge of the data-generating processes, ill-fitting priors are more easily identified both at the stage of manuscript review and the pre- and postreview stages. This is in contrast to current trends in frequentist inference where sampling intentions may not directly stated unless the study has been actively preregistered.

### Error quantification and model comparison

Critical to any statistical model and inference therein is its fit to observed data. While it is entirely possible to perform linear regression on data distributions that are highly nonlinear, predictions and inference made by the model will likely be inaccurate. Both Bayesian and frequentist inference offer robust model error quantification. Bayesian approaches, however, can utilize the posterior distribution to not only quantify and bound the distribution of model errors but also include post hoc posterior predictive sampling as part of the inference paradigm. Posterior predictive sampling involves making random draws from the posterior and building a sampling distribution. This distribution is then compared with the observed data distribution to quantify the model's disparity from observed data. Along with posterior predictive checks, prior predictive checks act as a sensitivity measure of the influence of the prior distribution on the posterior distribution. Taken together, Bayesian inference thus allows for robust statistical inference on observed experimental data which appropriately includes prior knowledge of the state of the field.

### Formulation of models and applied Bayesian inference

There are a multiplicity of programs and programming languages that facilitate Bayesian analysis, such as standalone programs like Jasp (Love et al., 2019) and probabilistic programming language packages such as BUGS (Brooks, 2003) and STAN (Carpenter et al., 2017). We chose to use PyMC (Salvatier et al., 2016) for its ease in explicitly declaring probability distributions and its implementation in Python, which is in common use in neuroscientific data analysis. Model formation is often conserved between frequentist and Bayesian approaches; it is only the mode of inference that differs. However, for clarity, we will discuss both model formation and performing inference in the subsequent sections.

## Materials and Methods

Bayesian inference was performed on a range of data typical to neuroscience experiments. Regression models, ANOVA models, and group comparisons are performed on single-unit activity recorded from IC neurons in response to auditory stimuli in young and aged rats (Palombi et al., 2001; Simon et al., 2004; Rabang et al., 2012; Herrmann et al., 2017; Bartlett et al., 2024). Random-effects regression models are performed on single units recorded in the auditory cortex (A1) using high-density recording arrays in response to infrared neural stimulation (INS; Izzo et al., 2007; Cayce et al., 2011, 2014; Coventry et al., 2024) of the medial geniculate body (MGB). All surgical procedures used in this study were approved by the Institutional Animal Care and Use Committee of Purdue University (West Lafayette, IN, #120400631) and in accordance with the guidelines of the American Association for Laboratory Animal Science and the National Institutes of Health guidelines for animal research. Sample sizes and subject details are given in their corresponding sections.

### Computational hardware

To underscore that meaningful Bayesian inference does not require cluster computing or extensive computational resources, all computations were performed on a Windows MSI GS-66 laptop with an Intel i7 central processing unit (CPU) with an Nvidia RTX2070 graphics processing unit (GPU). Our inference programs are CPU-bound, not requiring any GPU resources. Computations can be performed on most modern CPUs, but are accelerated with more CPU threads and cores and parallelization on GPUs.

### Bayesian linear regression and analysis of covariance assessment of the disruption of temporal processing in the IC due to aging

**Figure 3.**
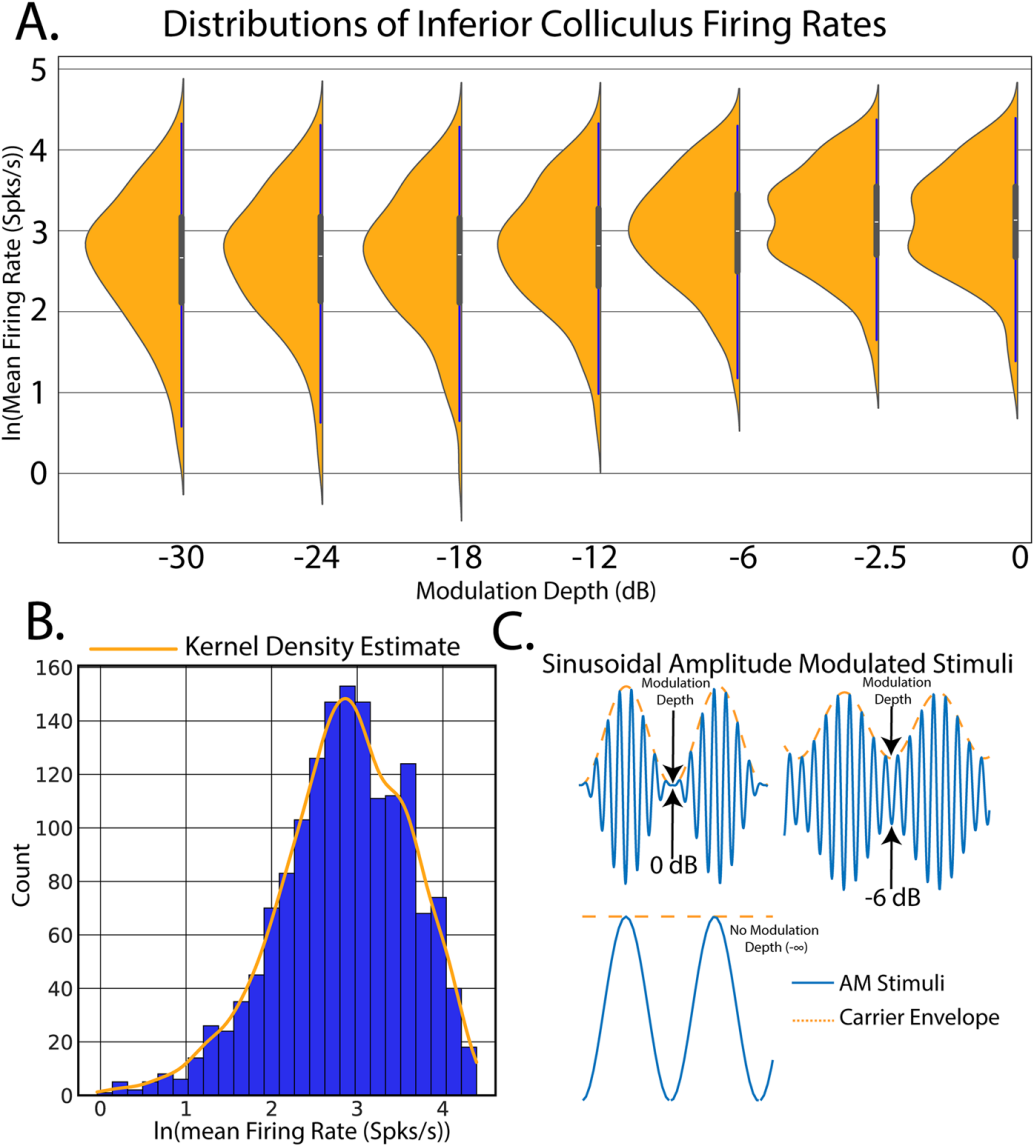
Example data for Bayesian simple linear regression on population estimates of firing rate versus amplitude modulation depth stimuli. Bayesian regression models were applied to population single-unit firing rates elicited from IC with SAM tones. The goal of this model was to predict evoked firing rates from increases in SAM modulation depths. ***A***, Density plot of observed firing rates versus SAM modulation depth. ***B***, Histogram and KDE of the distribution of SAM-evoked IC firing rates across all modulation depths. Observation of the total distribution of results aids in choosing the likelihood distribution for inference. ***C***, Schematic of amplitude-modulated stimuli.

The IC is the major integrative center of the auditory pathway, receiving excitatory inputs from the ventral and dorsal cochlear nuclei, excitatory and inhibitory inputs from the lateral and medial superior olivary complex (Kelly and Caspary, 2005), and inhibitory inputs from the superior paraolivary nucleus and the dorsal and ventral nuclei of the lateral lemniscus (Cant and Benson, 2006; Loftus et al., 2010). The IC encodes auditory information through hierarchical processing of input synaptics with local IC circuitry (Caspary et al., 2002; Rabang et al., 2012; Grimsley et al., 2013). Age-related changes in auditory processing primarily arise as deficits in temporal processing (Frisina and Frisina, 1997; Parthasarathy et al., 2010; Parthasarathy and Bartlett, 2012; Herrmann et al., 2017; Bartlett et al., 2024). This dataset is composed of single-unit responses recorded from 9 young (age ≤ 6 months) and 8 aged (age ≥ 22 months) male Fisher 344 rats. Auditory brainstem responses were recorded from animal subjects a few days prior to surgery to ensure hearing thresholds were typical of the rodent's age. Single-unit recordings were performed in a 9′ × 9′ double-walled, electrically isolated anechoic chamber (Industrial Acoustics). Animals were initially anesthetized via a bolus injection of ketamine (VetaKet, 60–80 mg/kg) and medetomidine (0.1–0.2 mg/kg) mixture via intramuscular injection. Oxygen was maintained via a manifold and pulse rate and blood oxygenation monitored through pulse oximetry. Supplemental doses of ketamine/medetomidine (20 mg/kg ketamine, 0.05 mg/kg medetomidine) were administered intramuscularly as required to maintain the surgical plane of anesthesia. An incision was made down midline and the skull exposed. Periosteum was resected and a stainless steel headpost was secured anterior to the bregma via three stainless steel bone screws. A craniectomy was made above the IC (−8.5 anterior/posterior and 1 mm medial/lateral from the bregma). A single tungsten electrode was advanced dorsally toward the central nucleus of the IC during which bandpass noise (200 ms, center frequencies 1–36 kHz in five steps per octave, 0.5 octave bandwidth) was delivered. The central nucleus of the IC was identified based on short-latency-driven responses to bandpass noise search stimuli with ascending tonotopy and narrowly tuned responses to pure tones of varying frequencies. Once neurons were identified, responses from 5 to 10 repetitions of sinusoidal amplitude-modulated (SAM) tones (750 ms tone length, modulation depth between −30 and 0 dB) were recorded using a preamplifying headstage (RA4PA, Tucker-Davis Technologies) and discretized at a sampling rate of 24.41 kHz (RZ-5, TDT). SAM tones were defined as follows:

$$s(t)=A[1+m\;*\;cos(2\pi f_{m}t+\varphi )]\;*\;n(t),$$

where *m* is the modulation depth ranging between 0.032,1(−30,0dB) , $f_{m}$ is the modulation frequency, *ϕ* is the reference phase of the modulator, *A* is the scaling factor for the stimulus sound level, and n(t) is the broadband noise stimulus. SAM stimuli are schematized in Figure 3*C*. Single units were filtered between 0.3 and 5 kHz. Offline spike sorting was performed using OpenExplorer (TDT).

### Bayesian multilinear regression assessment of thalamocortical recruitment from INS

INS is an optical technique that uses coherent infrared light to stimulate nerves and neurons without the need for genetic modification of the target or direct contact with tissue that offers spatially constrained activation above electrical stimulation (Wells et al., 2005; Izzo et al., 2007; Cayce et al., 2011, 2014; Coventry et al., 2020, 2024). In this study, seven male Sprague Dawley rats were chronically implanted in A1 with 16 channel planar Utah-style arrays (TDT) and stimulating optrodes in the MGB of the auditory thalamus (Thorlabs). Rodents were initially anesthetized with a bolus injection of a ketamine (80 mg/kg) and medetomidine (0.2 mg/kg) cocktail. Oxygen was maintained via a manifold and pulse rate and blood oxygenation monitored through pulse oximetry. Supplemental doses of ketamine/medetomidine (20 mg/kg ketamine, 0.05 mg/kg medetomidine) were administered intramuscularly as required to maintain the surgical plane of anesthesia. An incision was made down midline and the skull exposed. The periosteum was removed via blunt dissection, and three stainless steel bone screws were placed in the skull for headcap stability. An additional titanium bone crew was placed in the skull to serve as a chronic ground and reference point for recording electrodes. Craniectomies were made above the MGB (−6 anterior/posterior, −3.5 medial/lateral from the bregma) and auditory cortex (−6 anterior/posterior, −5 medial/lateral from the bregma). Fiber-optic-stimulating optrodes were placed in the midpoint of the MGB (−6 dorsal/ventral from the dura) and affixed to the skull using a UV-curable dental acrylic (Midwest Dental). A 16-recording channel planar array was putatively placed in layers 3/4 of the auditory cortex, with placement confirmed by short-latency high-amplitude multiunit activity elicited from bandpass noise (200 ms, center frequencies 1–36 kHz in five steps per octave, 0.5 octave bandwidth) test stimuli. Recording electrodes were sealed onto the headcap. Animals were allowed to recover for 72 h prior to the beginning of the recording regime. All recordings were performed in a 9′ × 9′ electrically isolated anechoic chamber. During recording periods, animals received an intramuscular injection of medetomidine (0.2 mg/kg) for sedation. Optical stimuli were delivered from a 1,907 nm diode laser (INSight open-source optical stimulation system) coupled to the optrode with a 200 µm, 0.22 NA fiber (Thorlabs FG200LCC). Laser stimuli were controlled via a RX-7 stimulator (TDT) and consisted of train stimuli with pulse widths between 0.2 and 10 ms, interstimulus intervals between 0.2 and 100 ms, and energy per pulse between 0 and 4 mJ. Applied laser energies were randomized to limit effects from neural adaptation with 30–60 repetitions per pulse width/interstimulus interval combinations. Signals from recording electrodes were amplified via a Medusa 32 channel preamplifier and discretized and sampled at 24.414 kHz with a RZ-2 biosignal processor and visualized using Open-Ex software (TDT). Action potentials were extracted from raw waveforms via real-time digital bandpass filtering with cutoff frequencies of 300–5,000 Hz. Single units were extracted offline via superparamagnetic clustering in WaveClus (Quiroga et al., 2004). Studies were performed to assess the dose–response profiles of optically based deep brain stimulation (DBS) over the span of several months. As each electrode recorded diverse populations of neurons which are potentially subject to change due to electrode healing in, age of the device, and adaptation to the stimulus, a within subjects, repeated measures regression model was warranted. Bayesian hierarchical regressions can easily deal with complex models such as these. This data was part of a previous study (Coventry et al., 2024).

### Bayesian *t* test assessment of computational models of BGTC function in Parkinson's disease

Parkinson's disease is a chronic and progressive neurological disorder resulting from a loss of dopaminergic neurons in the substantia nigra of the basal ganglia circuit (Dickson, 2018; Bove and Travagli, 2019). DBS, a technique by which therapeutic electrical currents are delivered to subthalamic neural structures using chronically implanted electrodes, has arisen as a potent treatment for motor symptoms of Parkinson's disease (Cagnan et al., 2019; Guidetti et al., 2021). However, the neural mechanisms underlying DBS remain unclear (Neumann et al., 2023). Computational models of the BGTC function in response to DBS provide insight into alterations of circuit dynamics as well as potential therapeutic targets. In prior work, a modified human mean-field BGTC model (Grado et al., 2018; Coventry and Bartlett, 2023) was implemented to study network DBS-encoding mechanisms in dopamine-depleted states. In the case of BGTC circuits, mean-field modeling consists of the average extracellular electric field response of collections of neurons with cellular properties modeled after each stage of the BGTC circuit in both healthy and dopamine-depleted states (Van Albada and Robinson, 2009). In each model trial, LFPs were recorded from the globus pallidus internus in resting state and stimulation conditions. Stimulation trials consisted of subthreshold (0.01 mA, 100 µs pulse width, 130 Hz) or suprathreshold (1.8 mA, 240 µs pulse width, 130 Hz) DBS of the subthalamic nucleus. The LFP activity in the β-band (13–30 Hz) is a known biomarker for Parkinson's symptomology (Little et al., 2013; Guidetti et al., 2021). As such, LFP power spectral density estimates of the β-band activity were calculated. The total LFP power in the β-band was calculated as follows:

$$P_{\mathrm{Tot}}=2\overset{30}{\underset{\;f=13}{\sum }}\;P_{xx}(f)\Delta f,$$

where $P_{xx}$ is the power spectral density at frequency *f*, Δf is the reflecting model sampling rate, and the factor 2 accounts for the frequency folding from Fourier decomposition.

### Inference reporting guidelines

Proper presentation of statistical methods and processes is critical to the interpretation of conclusions drawn from inference. While there are many reporting guidelines for Bayesian inference, we follow the Bayesian analysis reporting guidelines as given by Kruschke (2021) and provide an example reporting document including posterior predictive checks, Bayesian model comparisons, and sensitivity analysis in our GitHub code repository.

## Results

### Estimation of spike rates from auditory stimuli: a motivating example

To facilitate the discussion of Bayesian inference in neuroscience, consider an example found prominently in auditory neuroscience (Fig. 3). In our first experiment, single-unit recordings were made from the IC in response to applied SAM tones (see Materials and Methods). The goal of this analysis is to create a linear model of SAM temporal auditory processing by quantifying increases in evoked single-unit firing rates in response to decreased SAM modulation depth.

The linear regression model seeks to estimate a linear relationship between one (simple linear) or more (multilinear) predictor and measured variables. In this model, both the measured result and predictors are metric variables that map to a continuum of possible values, as opposted to categorical variables mapping to discrete classes. The simple linear regression model takes the form as follows:

y=α+βx+ϵ,

where y is the measured (predicted) group; x is the predictor; β is the “slope” parameter dictating the relative increase or decrease in y per unit change in x; α is the intercept term that, in models of firing rate, represents nonevoked, spontaneous firing rates; and ϵ is an error term that quantifies the difference between the expected value of y at a given x given a linear model versus the observed value of y at x. It should be noted that ϵ is not present in all regression models, but the authors suggest inclusion to quantify deviations from linear fit. Good practice in the analysis of data involves plotting distributions of responses in response to the stimulation variable (Fig. 3*A*) and the distributions of data across all stimulation conditions (Fig. 3*B*).

**Figure 4.**
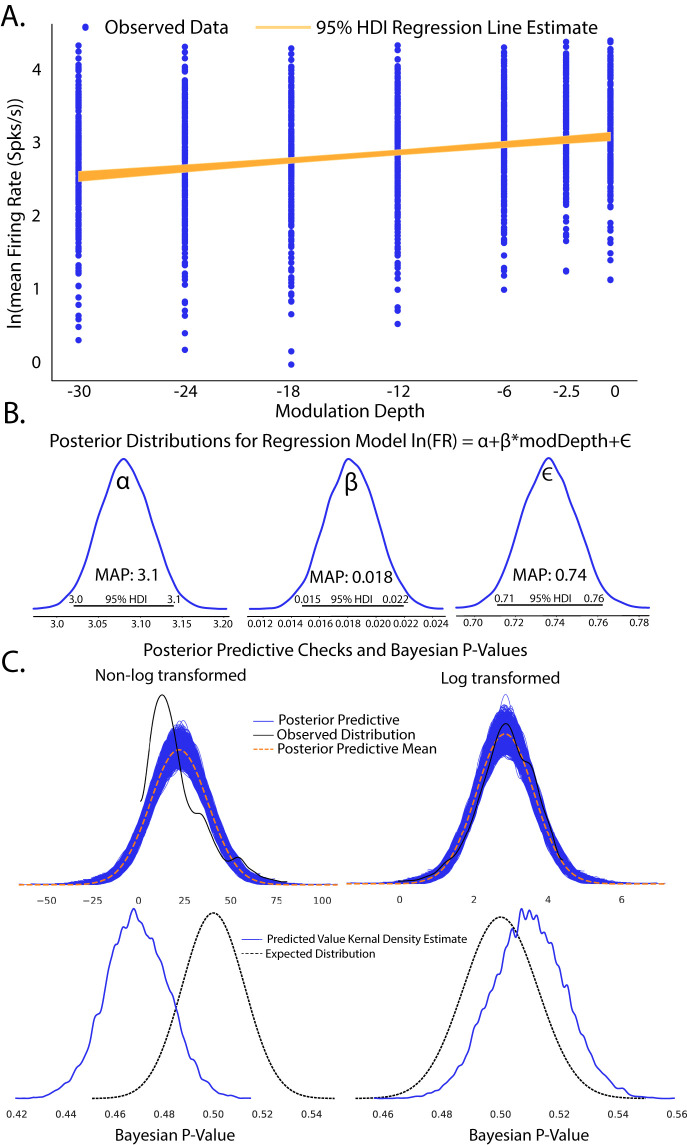
Completed Bayesian inference quantifying linear relationships in evoked firing rate from increases in modulation depth. ***A***, Scatter plot of observed firing rates versus SAM depth stimuli with fitted regression line estimates superimposed. Ninety-five percent HDI estimates of regression slopes are shown in orange, with the spread of lines encoding the 95th percentile of most likely slope values. ***B***, Estimates of Bayesian linear regression parameters. Intercept term α was significantly above 0 (MAP = 3.1; 95%HDI does not overlap 0) which indicates basal firing rates above 0. Regression slope was small but significantly above 0 (MAP = 0.018; 95% HDI does not overlap 0), suggesting an increase in evoked firing rates with increased modulation depth. Error term ϵ was significantly above 0 (MAP = 0.74; 95% HDI does not overlap 0), suggesting some model deviation from observed data. However, error terms were considered small as ϵ MAP <α basal firing rate MAPs. ***C***, Posterior predictive checks of linear (left) and log linear (right) regression models show that log-transformed firing rate models produce posterior predictions most inline with observed data. Disparity of empirical posterior predictive distributions from observed data as quantified through Bayesian *p*-values also suggests that log-transformed firing rates create a superior model fit. Dashed black lines represent the expected distribution for a dataset of the same size as the observed neural dataset. The solid blue line denotes the kernel density estimate of the proportion of posterior-predicted values that are less than or equal to the observed data.

Linear regression forms a model in which SAM depth predicts evoked firing rates in which the model parameters are estimated and used to draw conclusions about the relative dependency of y on x. To begin, an observation of the distribution of the measured data, in this case firing rates elicited from the IC, will allow for robust inference model design. Data distributions are most often visualized through construction of data histograms. Pdfs can then be estimated through kernel density estimation (KDE), a process in which data points are convolved with Gaussian kernels to create a smooth estimation of underlying continuous data pdfs (Rosenblatt, 1956; Parzen, 1962). Inspection of the distribution of firing rates (Fig. 3*B*) suggests that a log transform would allow for the data to be normally distributed, making model computations easier through the use of canonical normal distributions. Observation of total data pdfs also guides the choice of the likelihood distribution which describes the data-generating process. After natural log transformation, data pdfs approximate a normal distribution, making a normally distributed likelihood function a good choice for regression inference. This choice, however, should be validated using posterior predictive checking, which is shown after performing Bayesian inference using the linear regression model (example shown in Fig. 4*C*).

### Performing Bayesian inference on the linear regression model

Turning back to the example of IC single-unit firing rates in response to SAM depth stimuli, the first step in inference is to place a prior distribution on the data. Previous studies and data can be used to inform the prior, but for this example, we chose to demonstrate regression with moderately informative priors on α,β, and ϵ so as to let observed data drive posterior inference. Given that the observed data is roughly normal, a good first pass is to place a normal distribution on the prior with mean equal to the mean of the observed data and a variance that is wide enough to capture all observed data. After inference is made, sensitivity analyses can be performed to assess the relative importance of the prior parameter values on posterior estimates. Larger prior variances allow for small, but nonzero, probabilities on extreme values. This tends to be a more robust approach than setting a value of 0 on extreme events, as observed data with strong evidence for an extreme value can be adequately represented in the posterior. After observation of the underlying distribution of the observed data and decision on a prior distribution, a linear regression inference model can be easily described in code as follows:

Code Example 1: PyMC initialization of a simple linear regression model

**Figure:**
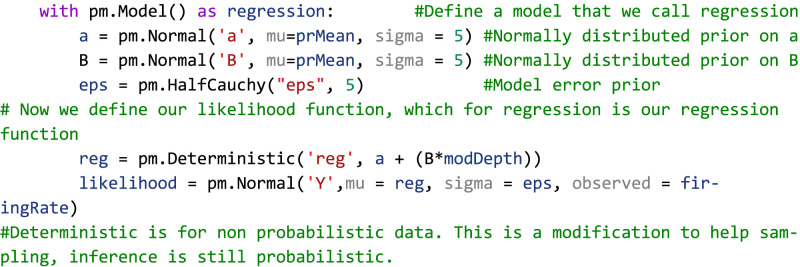

The likelihood variable then translates our model to one of the Bayesian inferences by casting the model as a probability distribution, in this case, as follows:

y∼N(α+βx+ϵ).

Importantly, Bayesian inference does not require any extra preprocessing of the data outside of what is dictated by the experimental design. In this case, we are interested in assessing the interplay of varying modulation depth on maximum firing rates. Our raw data then is estimated mean firing rates calculated from peristimulus time histograms (PSTH), which are incorporated into the regression model in the “observed” variable in the likelihood function. To generate the posterior, all that needs to be done is to initialize and run the MCMC as follows:

Code Example 2: running the MCMC sampler

**Figure:**
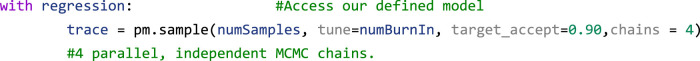

This routine calculates the regression model, generating a trace variable containing the posterior distributions of all model parameters after sampling numSamples with numBurnIn samples to initialize chains. We also ran four chains in parallel with a target_accept probability of 90%. Acceptance probability is based on the statistics of observed data and model, with more difficult posteriors benefiting from higher accept probability values (Gilks et al., 1996). Improper acceptance probabilities can give rise to insufficient number of draws and malformation of posterior distributions. PyMC provides a helpful readout for when posterior draws are malignant and indicative of higher acceptance probabilities. In summary, in a few lines of code, the researcher has observed distributions of the data and explicitly defined a model of the data generator and likely now has a better intuition of the data and how it is distributed. All that is left to observe the posteriors with HDIs to infer significance from the model.

Plotting the 95% HDI estimation of the regression line (Fig. 4*A*) of modulation depth versus natural log-transformed firing rates suggests a small but significant increase in firing rates with increases in modulation depth as evidenced by a 95% HDI credible region (0.015–0.022) that does not include zero from the β posterior distribution (Fig. 4*B*). A MAP value of 0.018 represents the most probable slope value from the regression. Posterior distributions of model parameters (Fig. 4*B*) also show that there is an estimated basal firing rate above 0 (α MAP = 3.1) with model error terms considered small for being significantly smaller than the intercept term ( ∈ MAP = 0.74). The spread of the 95% HDI on inferred parameters is used as a measure of uncertainty of the parameter, with narrow HDIs representing more certainty in the MAP estimated parameter. In our model, the α parameter has a spread between 3.02 and 3.13, with a difference of 0.11 containing 95% of its posterior distribution, suggesting strong certainty in the MAP estimate of 3.1. A similar narrow spread is seen in the β parameter, with a difference of 0.007 containing 95% of the posterior. The model error term shows that the observed data deviation from the model is constrained between 0.71 and 0.76, suggesting relative certainty in the magnitude of deviation of the data from the model. In the context of regression, these posterior results can be interpreted as a mean firing rate increase by a factor of 1.02 (2%, $e^{0.018}$) per percentage change in SAM depth that follows a most probable model of the following:

ln(meanFiringRate)=3.1+0.018*modDepth+0.74.

The ∝ term in the context of this study represents the natural log firing rate for 0% modulation, corresponding to a pure tone stimulus. Statistical conclusions should not end after making inferences on model parameters, however. Critical to the validity of statistical inference is the quality of the model fit to observed data. This goodness of fit in Bayesian approaches can be analyzed by posterior predictive checks, in which sample draws are made from the posterior distribution, simulating observations of data generated from the experiment from which the statistical model was fit, and comparing sampled to observed data to assess deviation of model predictions from observed data distributions. In PyMC, posterior predictive checks can be easily performed using the following code:

Code Example 3: performing posterior predictive checks

**Figure:**
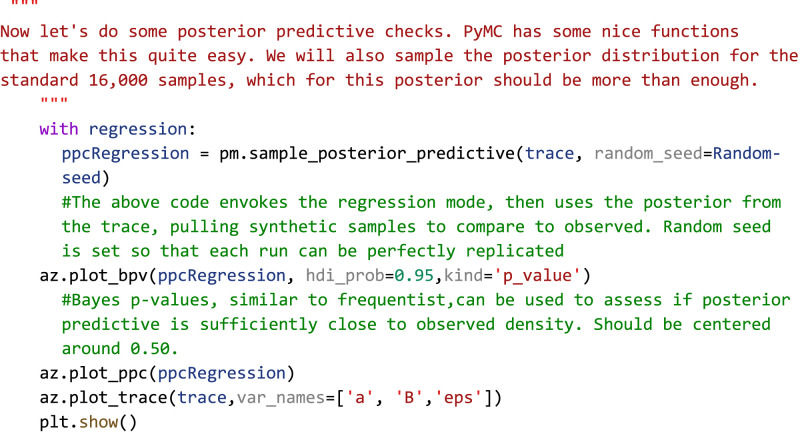

To illustrate how posterior predictive checks can be used, a competing model was made which performs Bayesian linear regression fit to the same data and priors except without log transformation of the data. In each case, random draws were made from each log-transformed and nonlog-transformed posterior to create empirical data distributions. Comparison of empirical distributions qualitatively shows that log-transformed models present a better fit to observed data than nonlog-transformed models. The relative disparity between posterior predictive model fits and observed data can be quantified by the use of Bayesian *p*-values (Fig. 4*C*), a distance measure between two distributions (for details of Bayesian *p*-values, see Kruschke, 2014). The closer the Bayesian *p*-value is to 0.5, the better data sampled from the posterior overlaps with the distribution of observed data. Plotting the resulting distributions and the Bayesian *p*-values indeed shows the log-transformed model fits better to observed data than the nontransformed model. Similar analyses can be performed around model-free parameters, such as prior variables, to form a sensitivity analysis of our prior distributions on resulting posterior inferences.

**Figure 5.**
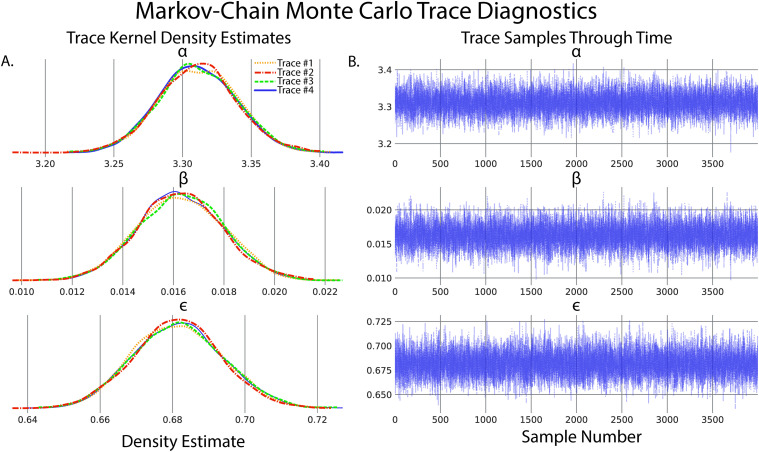
Evaluation of MCMC chains can help diagnose ill-fitting distributions. ***A***, Kernel density estimates of the marginal posteriors corresponding to each of the regression parameters of each MCMC trace. Qualitatively, chain distributions should appear similar to each other, suggesting good convergence to target distributions. ***B***, Time-series plot of trace value versus sample number of marginal posteriors corresponding to each regression parameter. Qualitatively, good traces should have a “fuzzy caterpillar”-like shape, evident in all parameters of this model, indicative of good integration over the joint posterior distribution and effective sampling of the posterior.

A secondary and quick check of posterior sampling can be performed by qualitative evaluation of the MCMC sampling chains, often called traces. Traces represent the long-term run of a Markov chain which represents the distribution of interest. As such, good traces show evidence of effective sampling and convergence to target probability distributions. PyMC offers easy ways to visualize posterior MCMC traces using the plot_trace function. Figure 5 shows traces obtained from our Bayesian regression example. The kernel density estimates of traces corresponding to the posterior distributions of regression parameters show good convergence of MCMC traces to a target distribution (Fig. 5*A*). As MCMC chains are time-series samples that form a distribution, evaluation of traces through sampling time can also be used as a diagnostic of sampling convergence. Traces should have a “fuzzy caterpillar”-like appearance (Fig. 5*B*) without any stark jump discontinuities from sample to sample. Quantitative trace evaluations are also available, with the Gelman–Rubin statistic $\left(\hat{r}\right)$ being the most prominent. The Gelman–Rubin statistic measures the variance between MCMC chains to the within-chain variance, effectively measuring chain stationarity and convergence (Gelman and Rubin, 1992). Heuristically, $\hat{r}<1.05$ is considered good convergence of MCMC chains. This value can be calculated *post hoc* after sampling, and PyMC will automatically flag if $\hat{r}\geq 1.05$ is detected.

### Comparison to frequentist linear regression

We next applied frequentist ordinary least squares (OLS) regression to the above log-transformed data implemented in the SPSS software (IBM). OLS regression produced point estimates on model parameters are very similar to what was found in Bayesian regressions, with increases in modulation depth resulting in increased evoked log firing rates (β=0.018;p<0.01). Regression intercept was also statistically significant and close to what was found in Bayesian regression (α=3.082;p<0.01). This agreement is not surprising, as first-order, point model estimates in both frequentist and Bayesian inferences with minimally informative priors often agree, especially in low variance data with stronger effects (Ruli and Ventura, 2021). However, frequentist regression only offers point estimates for regression parameters, so the resulting inference tends to be dichotomized to results being significant or not significant. Bayesian inference, on the other hand, not only offers a point estimate of the most likely parameter value in the MAP estimate but also offers a direct probability estimate of the credibility of the model given observed data in the 95% HDI and credible intervals, allowing for explicit quantification of the probability of the significance of the model parameter θ given the data observed directly. As an example, consider the posterior distribution of the modulation depth slope parameter β, the 95% HDI, and the corresponding credible interval (0.015,0.022) and MAP estimate 0.018 (Fig. 4*B*). The MAP value directly gives the most likely parameter estimate and observation of the scale of the effect. The credible interval then adds to the point MAP estimate by showing other probable slope values given the observed data. Given what is known about the neurons of the IC, this spread suggests some heterogeneity in neural response to changes in SAM stimuli modulation depth, generating hypotheses for future study. However, because the relative spread of the credible interval is small, any heterogeneity found in the IC response is likely to be small in variance. Furthermore, because the slope parameter value corresponding to no effect (β=0) is not in the 95% HDI and is well in the tail of the posterior distribution, the probability of modulation depth with no effect on IC firing rates is vanishingly small. Finally, direct observation of the posterior and underlying data from posterior predictive checking immediately validates that the statistical model from which inference is drawn is a good fit to the data collected (Fig. 4*C*). Frequentist confidence intervals offer a similar inference modality to the credible interval. However, confidence intervals are not themselves distributions and do not directly assess the probability of the parameter θ given the observed data. Rather, confidence intervals are dependent on a potentially unobserved population distribution. Efforts are being made to create so-called “confidence distributions,” aiming to provide similar interpretability to Bayesian credible intervals in frequentist contexts (Xie and Singh, 2013), but confidence distributions are still nascent and not yet fully developed.

In many cases, frequentist paradigms are used to generate binary inferences (significant/nonsignificant) without consideration of the underlying data distribution or fit of model to the observed data. As an example, consider the non-log-transformed linear regression shown in Figure 4, *B* and *C*. OLS regression gives a significant result (β=0.318;p<0.001). However, observation of the underlying data suggests that this does not fit a normal distribution, an underlying assumption of OLS regression. It would be tempting to stop when a significant *p*-value is met without considering how well the statistical model fits to observed data. An advantage of Bayesian approaches is that observation of posterior and data distributions is a requisite for inference, in which bad model fits to data are more easily observed.

Finally, we note that our OLS regression should be treated as a *post hoc* analysis, as sampling intentions were not planned ahead of time, and thus subject to statistical biases inherent to performing frequentist analyses without the proper pre-experimental planning stage formalized in the preregistration of studies.

### *T* tests and comparison of groups

Comparison of differences between two or more groups is one of the most fundamental inference paradigms, ubiquitous to all fields of neuroscience. Frequentist implementations of group comparisons, such as the *t* test and $\chi^{2}$ tests, suffer from similar ailments as frequentist regressions: strict assumptions about distributions of observed data, lack of interpretability of group differences, and the inability to confirm a null hypothesis. Added to these is that of the multiple-comparisons problem, with increasing number of comparisons leading to increasing Type I errors. Bayesian implementations of group comparisons provide complete descriptions of group differences in means, variances, and effect sizes, the ability to confirm and not simply reject null hypotheses, and complete posterior distributions, which remove the need for multiple-comparisons corrections (Gelman et al., 2009; Kruschke, 2011, 2013). One implementation of Bayesian group comparisons is Bayesian estimation supersedes the *t* test (BEST) implemented and described in Kruschke (2013), consisting of a hierarchical model with data described by *t* distributions and minimally informative priors on group means and standard deviations. Thus, the BEST model serves as the Bayesian analog to the frequentist *t* test.

### Comparison of β-band dynamics in a DBS model using BEST

**Figure 6.**
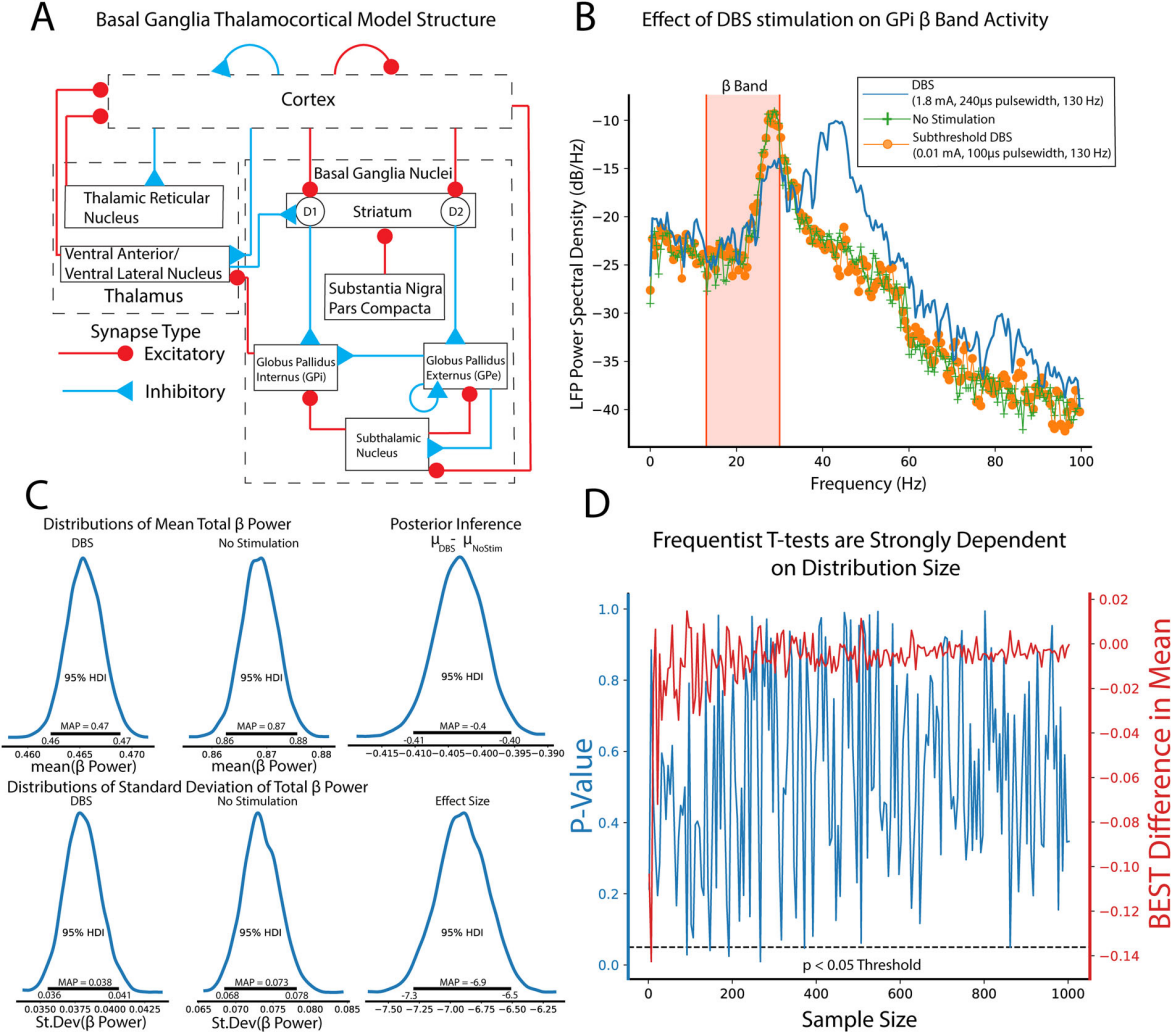
Bayesian implementations of *t* tests provide descriptive quantification of differences in group means, standard deviations, and effect sizes drawn directly from observed data. ***A***, Schematic of the basal ganglia–thalamocortical computational model. Modeled DBS stimulation was performed in the subthalamic nucleus with LFP recordings made from the globus pallidus internus. All simulations were performed assuming dopamine-depleted conditions. ***B***, Example LFP power spectral densities from no stimulation condition (green), subthreshold DBS stimulation (0.01 mA, 100 µs pulse widths, 130 Hz, orange), and effective DBS stimulation (1.8 mA, 240 µs pulse widths, 130 Hz, blue). ***C***, Total β-band power in no stimulation and DBS stimulation was calculated (*n* = 2,000 repetitions). Group comparison by BEST shows significant reduction in mean β-band power as evidenced by group mean difference 95% HDIs not including 0 (MAP $\mu_{\mathrm{DBS}}-\mu_{\mathrm{nostim}}=-0.4).$ Estimated effect size distributions further give evidence of a strong DBS effect of lowering β-band power in dopamine-depleted conditions (MAP_effect size_ = 6.9; 95% HDI does not contain 0). ***D***, Frequentist *t* tests show strong dependence on the sample size. Comparisons of β-band power were made between no stimulation and subthreshold stimulation groups. Subthreshold stimulation will have no effect on β-band power with only differences formed by model noise simulating intrinsic neural noise. Increasing the numbers of shuffled samples were included in *t* test and BEST tests. BEST differences in group means show rapid convergence to null differences. *P*-values from frequentist *t* tests however show drastic fluctuations, some of which cross p<0.05 significance threshold even at large sample sizes.

To illustrate the use of BEST in group comparisons, a computational model of the dopamine-depleted BGTC circuit was utilized (Fig. 6*A*). Simulations consisted of measurements of oscillatory activity in LFP β-band (13–30 Hz), elevation of which is a known biomarker for parkinsonian symptomology across the basal ganglia circuit (Singh, 2018). No stimulation, subthreshold “sham” stimulation, and effective DBS stimulation groups were tested and compared. LFPs were recorded from the globus pallidus internus and the subthalamic nucleus was stimulated with 2,000 repetitions of each stimulation group. Figure 6*B* shows example LFP power spectrums for no stimulation, subthreshold stimulation, and DBS conditions, respectively, with reductions in β-band activity shown in DBS conditions compared with no stimulation and subthreshold stimulation. Posterior distributions allow for estimates of group means and standard deviations of LFP β-power (Fig. 6*C*) with qualitative observations of nonoverlapping credible regions of means (DBS, 0.46–0.47; no stimulation, 0.86–0.88) and standard deviations (DBS, 0.036–0.041; no stimulation, 0.068–0.078) suggesting that DBS produces strong reductions in β-power. Unlike frequentist methods, direct measurements of differences in group means are easily quantified from subtraction of group posterior distributions. We reiterate that unlike frequentist methods, this inference is formed directly from observed data, and not conditioned on hypothetical population distributions. Differences in group means show strong reductions in β-band power in DBS conditions (MAP estimate, $\mu_{\mathrm{DBS}}-\mu_{\mathrm{nostim}}=-0.4$) with MAP estimates of effect size showing strong, discernible evidence of the DBS effect in reducing β-oscillatory activity (MAP = −6.9). Differences in group means and effect size are considered significant because 95% HDIs do not contain 0.

Estimation of group differences further shows the explanatory power of Bayesian inference in its ability to provide direct, probabilistic estimates of group summary statistics, group differences, and effect sizes. Bayesian approaches with appropriate priors are also more robust to sample size, particularly lower sample sizes that are typical of many neuroscience experiments (Smid et al., 2020). To illustrate this, we utilized frequentist *t* test and BEST inference, comparing no stimulation and subthreshold stimulation groups. BEST and *t* test estimates on these groups should confirm a null hypothesis/fail to reject the null hypothesis for Bayesian and frequentist inference respectively given no effect of subthreshold stimulation on LFP β-band activity. In this study, random study stopping points were simulated by performing BEST and *t* test estimates on increasing sample sizes (2–1,000, steps of 5). Data points were pulled from random permutation of no stimulation and subthreshold stimulation groups. BEST estimation showed rapid convergence to estimated group differences of 0 at low sample sizes, while frequentist *p*-values showed strong fluctuations regardless of sample size (Fig. 6*D*). *P*-value oscillations showed several instances of falling below p<0.05 decision criteria, even at sample sizes larger than the majority of neuroscientific experiments. While *p*-value dynamics are contextual, based on data type and underlying data distributions, this data shows the potential of NSHT to draw drastically different experimental conclusions from sampled data distributions with very similar sample sizes.

### Multilinear regressions, repeated measures, and hierarchical models

In many experiments, inference across multiple possible data-generating parameters must be analyzed and accounted for. These models, called multilinear regressions, are extensions of standard linear regression as follows:

$$y=X^{T}\beta +\epsilon \to y=\;\beta_{0}+\beta_{1}x_{1}+\beta_{2}x_{2}\ldots +\beta_{n}x_{n}+\epsilon ,$$

where *n* is the total number of predictors and β terms corresponding to slope terms of each predictor variable.

**Figure 7.**
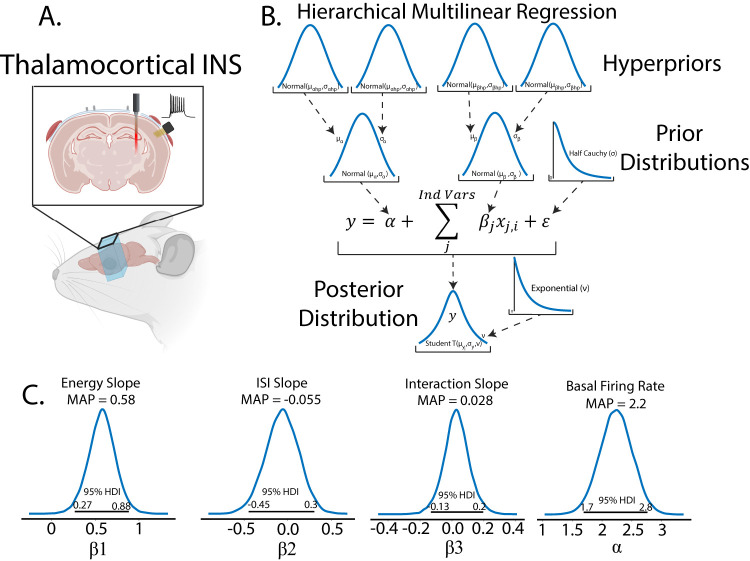
Example of Bayesian multilinear regression incorporating a hierarchical structure. ***A***, In this experiment, rodents were implanted with fiber-optic arrays into the auditory thalamus and planar recording arrays into the auditory cortex. Single-unit responses were recorded from INS stimuli with applied energy and interstimulus intervals varied to derive dose–response curves. Figure was drawn using BioRender under publication license (www.biorender.com). ***B***, Hierarchical schematic of Bayesian multilinear regression. Hierarchical structures are advantageous in accounting for within- and between-subject variability or for repeated measures designs. ***C***, Resulting parameter distributions from dose–response models. Energy was a significant contributor to maximum firing rate, with increasing laser energy resulting in increased maximum firing rate, as determined by the 95% HDI of the laser energy term $\beta_{1}$ excluding 0 (MAP = 0.58). Laser pulse interstimulus interval did not significantly contribute to changes in maximum firing rate as indicated by ISI parameter $\beta_{2}$ overlapping 0 in its 95% HDI with a MAP value near 0 (MAP = 0.028). The relatively wide spread about zero does suggest that there may be a subset of ISIs that contribute more strongly to firing rates and warrant further study. Laser energy–ISI interactions also did not significantly contribute to maximum firing rate as evidenced by interaction parameter $\beta_{3}$ including 0 in its 95% HDI. The intercept term α, corresponding to basal firing rates, was significantly above 0 (MAP = 2.2; 95% HDI excludes 0).

To illustrate the use of multilinear regressions, consider the case of thalamocortical INS (Fig. 7*A*). Auditory thalamic neurons in the MGB were excited by pulse trains of optical stimuli varying in pulse energy and time between pulses. The resulting auditory cortex single-unit responses were recorded using a planar, Utah-style array in layer 3/4. An important and understudied aspect of INS is the effect of laser energy and interstimulus interval changes on evoked firing rate responses quantified by a dose–response curve. We begin by specifying predicted and predictor values. Dose–response relationships were measured by predicting maximum firing rates in response to applied INS energy (E) and interpulse intervals (ISI). As we suspect an interaction between E and ISI, an interaction term of E * ISI was incorporated. Therefore, the model was defined as follows:

$$\max(\mathrm{FR})=\;\alpha +\beta_{1}E+\beta_{2}\mathrm{ISI}+\;\beta_{3}(E\;*\;\mathrm{ISI})+\epsilon .$$

An important aspect of this study was that rats underwent chronic recordings through the duration of the lifetime of the implant. It almost a certainty that stimulation and recording quality will change over the lifetime of the devices due to neural adaptation to stimulation (Falowski et al., 2011) and glial response and encapsulation of the devices (Van Kuyck et al., 2007; Woolley et al., 2013). This experimental paradigm is thus complicated by potentially meaningful repeated measures within-subject variability. Furthermore, slight differences in electrode and optrode placement between rodents could create a heterogeneity in the receptive fields of recorded neurons (Vasquez-Lopez et al., 2017), representing a potentially meaningful between-subject variance.

### Hierarchical structures capture latent variables

Models in both Bayesian and frequentist paradigms capture these within- and between-subject variances by adding hierarchical structure to the model. From the Bayesian perspective, hierarchical models are defined by allocating hyperparameters on the prior which encode within- and between-group variances in the model, with each hyperparameter containing hyperprior distributions. Graphically, this is organized in Figure 7*B*. Bayesian and frequentist hierarchical models share similar roots, with particular hyperprior distributions in Bayesian paradigms becoming proportional to frequentist random effects models.

While this appears to be a herculean task in data modeling, PyMC allows for declarations of hierarchical models, as shown in Code Snippet 4:

Code Example 4: creating a hierarchical regression model

**Figure:**
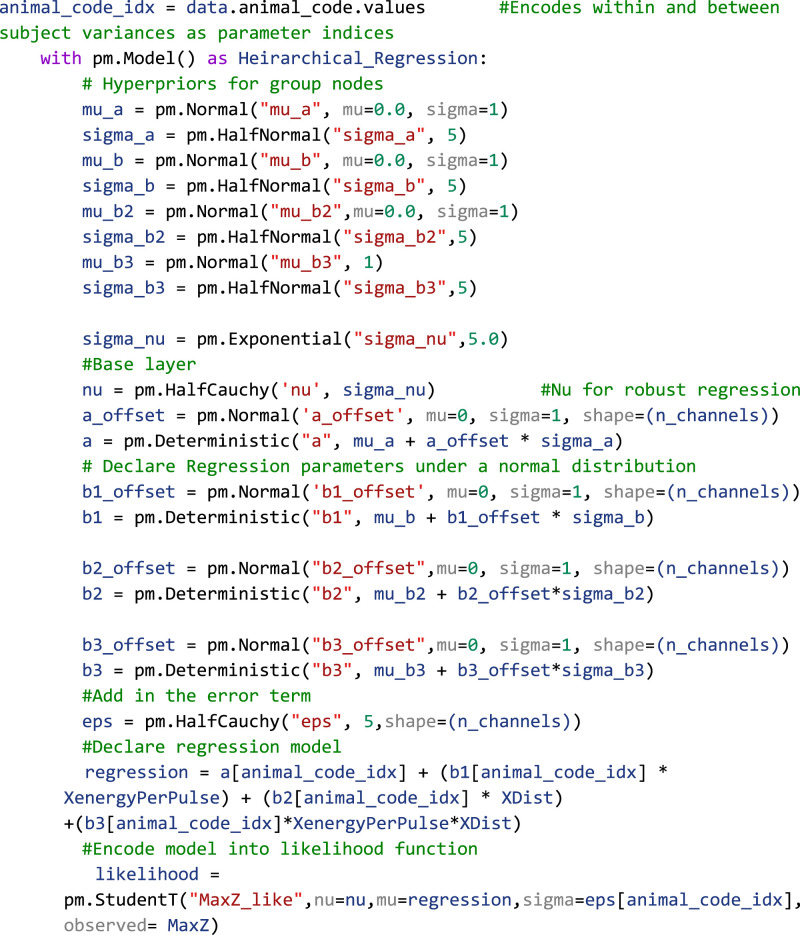

Owing to the scarcity of thalamocortical INS data, we assigned noninformative, widespread normal distributions on the priors and hyperpriors so as to let the data speak for itself. We also utilized a Student’s *t* distribution as the likelihood function to accommodate outliers in a modification known as robust regression (Kruschke, 2014). Student’s *t* distributions have tails that are not bounded by the exponential function, meaning that extreme values have less impact or skew on the posterior distribution. Half-Cauchy distributions are placed on the error term and Student’s *t* normality parameter ν. Half-Cauchy distributions are advantageous in learning scale parameters from the data in hierarchical models (Gelman, 2006; Polson and Scott, 2012).

It is important to validate that our model and data-generating functions indeed represent the observed data. Sensitivity analyses and posterior predictive checks thus can be performed to ensure the model chosen is the one that best describes the observed data. Sensitivity analyses were performed by varying prior variance and comparing models that were nominal or natural log transformed with normal (N) and Student’s *t* (ST) likelihood functions. Model comparisons can be performed in many ways, but a common paradigm is the leave-one-out cross-validation (LOO; Gelman et al., 2014). LOO consists of partitioning data into training and test sets and iteratively fitting the model under test with training data and testing out-of-sample fits with test data. Models are then ranked using the expected log pointwise predictive density (ELPD) measure:

$$\mathrm{ELPD}=\;\overset{k}{\underset{i=1}{\sum }}\;\int dy_{i}p_{t}\bar{y_{i}}\log(\;p(\bar{y_{i}}|y)),$$

where $p_{t},y_{i}$ are unknown distributions representing the true data-generating function for estimates of true posterior predictive function $\left(\bar{y}|y\right)$ from the observed data y (Vehtari et al., 2017). In general, larger values of ELPD represent better out-of-sample fits indicative of a better model conditioned on observed data. We can then use standard errors between the model with the best ELPD (dse) and all competing models to rank all models to observed data. Importantly, these metrics should be understood only in the context of a model relative to other models, and not a global predictor of model validity. Observations of posterior fits to the data using posterior predictive fits and Bayesian *p*-values should be utilized on the final model to determine model fit. This seemingly complex model comparison can be quickly and easily done in PyMC with the following commands:

Code Example 5: model comparisons

**Figure:**
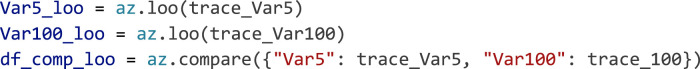

Model comparison results are given in Table 2. Similar to the simple regression above, the log-transformed model provided much better fits to observed data than nonlog-transformed models. Interestingly and instructively, moderately informative priors (variance 5) outperformed noninformative priors (variance 100), suggesting that constraining prior variance can have predictive power in inference. Posterior predictive checks on the winning model show good fits to observed data with a Bayesian *p*-value near 0.5.

**Table 2.** LOO model comparisons and sensitivity analyses

Model	R	ELPD	DSE
St Log Var 5	1	−5,337.48	0.00
ST Log Var 100	2	−5,337.62	0.420867
ST Log Var 0.5	3	−5,337.76	0.409773
St Log Var 25	4	−5,338.15	0.492297
ST Log Var 10	5	−5,338.18	0.300197
ST Log Var 1	6	−5,338.26	0.331152
N Log Var 10	7	−5,340.60	3.308668
N Log Var 1	8	−5,341.09	3.293779
N Log Var 5	9	−5,341.16	3.296273
N Log Var 0.5	10	−5,342.46	3.300550
ST Semilog Var 1	11	−5,466.76	15.845916
St Semilog Var 5	12	−5,467.12	15.856552
ST Semilog Var 10	13	−5,467.15	15.895646
ST Semilog Var 0.5	14	−5,467.18	15.866405
ST Var 1	15	−15,336.31	79.406629
ST Var 0.5	16	−15,355.67	80.415787
St Var 5	17	−15,355.67	80.415787
N Var 10	18	−16,119.11	82.384329
N Var 1	19	−16,132.23	83.549811
N Var 0.5	20	−16,154.55	84.262219

We can now perform inference on our multiregression model. It was found (Fig. 7*C*) that α was significantly above 0 (MAP = 2.2; 95% HDI does not cross 0) suggesting that basal firing rates of recorded neurons were typically above 0 as expected. It was also seen that maximal firing rates were significantly dependent on the applied INS energy ( $\beta_{1}$MAP = 0.58; HDI does not contain 0) with increases in INS energy leading to larger evoked maximal firing rates. The relative spread of the 95% HDI on $\beta_{1}$ of 0.27–0.88 suggests a heterogeneity in neuron dose–response characteristics that can be subsequently explored in future studies. Somewhat surprisingly, there was no significant effect of ISI on maximum firing rates ( $\beta_{2}$ MAP = −0.055). The relative spread across 0 of −0.45 to 0.3 suggests that extreme values of ISI might potentially have an effect, with smaller ISIs causing neural integration of singular INS pulses into a singular, large pulse. However, that cannot be determined given the INS parameters used in this study. Also surprisingly, there was no significant effect of energy–ISI interactions ( $\beta_{3}$ MAP = 0.028), suggesting that INS energy is the primary mediator of evoked firing rates.

### Bayesian ANOVAs

Comparison of differences between groups is another routine statistical procedure used when predictor variables are nominal or categorical in nature or a mixture of metric and categorical predictors. The frequentist treatment of these experimental designs largely uses analysis of variance methods, namely, ANOVA for categorical predictors and, more generally, analysis of covariances (ANCOVAs) for categorical predictors with metric covariates. ANOVAs are models that take the form of:

$$y=\;\alpha +\underset{i}{\sum }\;\beta_{i}x_{i},$$

where $\beta_{i},x_{i}$ are the parameters corresponding to nominal predictor class i, α is the offset or bias parameter, and y is the metric-dependent variable. ANOVA parameters and class values $\beta_{i},x_{i}$ are treated differently than the regression case, as $x_{i}$ are categorical as opposed to continuous, metric values, and as such x categories are recast into “one-hot” encoded vectors $\vec{x}=[x_{0},x_{1},\ldots ,x_{i}]$ in which only a singular value in an array can have a value of 1 and all other elements are cast to 0, allowing for binary indication of a given class among a group of classes. If an individual value falls into group j, for example, ${\vec{x}}_{i\neq j}=0,{\vec{x}}_{i=j}=1$. The coefficients $\beta_{i}$ then encode the change in dependent variable y from inclusion of datapoint x in category i. Importantly, deflections from baseline are constrained such that $\sum_{i}\beta_{i}=0$. Both Bayesian and frequentist ANOVA models treat $\beta_{i}$ parameters as group deflections about the baseline level of the dependent variable.

ANCOVA is a modification to the ANOVA model to include a metric covariance term:

$$y=\;\alpha +\underset{i}{\sum }\;\beta_{i}x_{i}+\beta_{\mathrm{cov}}\;x_{\mathrm{cov}}$$

where $\beta_{\mathrm{co}},x_{\mathrm{co}}$ are the parameters corresponding to metric predictors. Metric predictor terms are valuable in accounting for within-group variance, which is attributable to some other metric measurable variable, such as decreased firing rates in response to an applied stimulus found in a class of aged animals.

**Figure 8.**
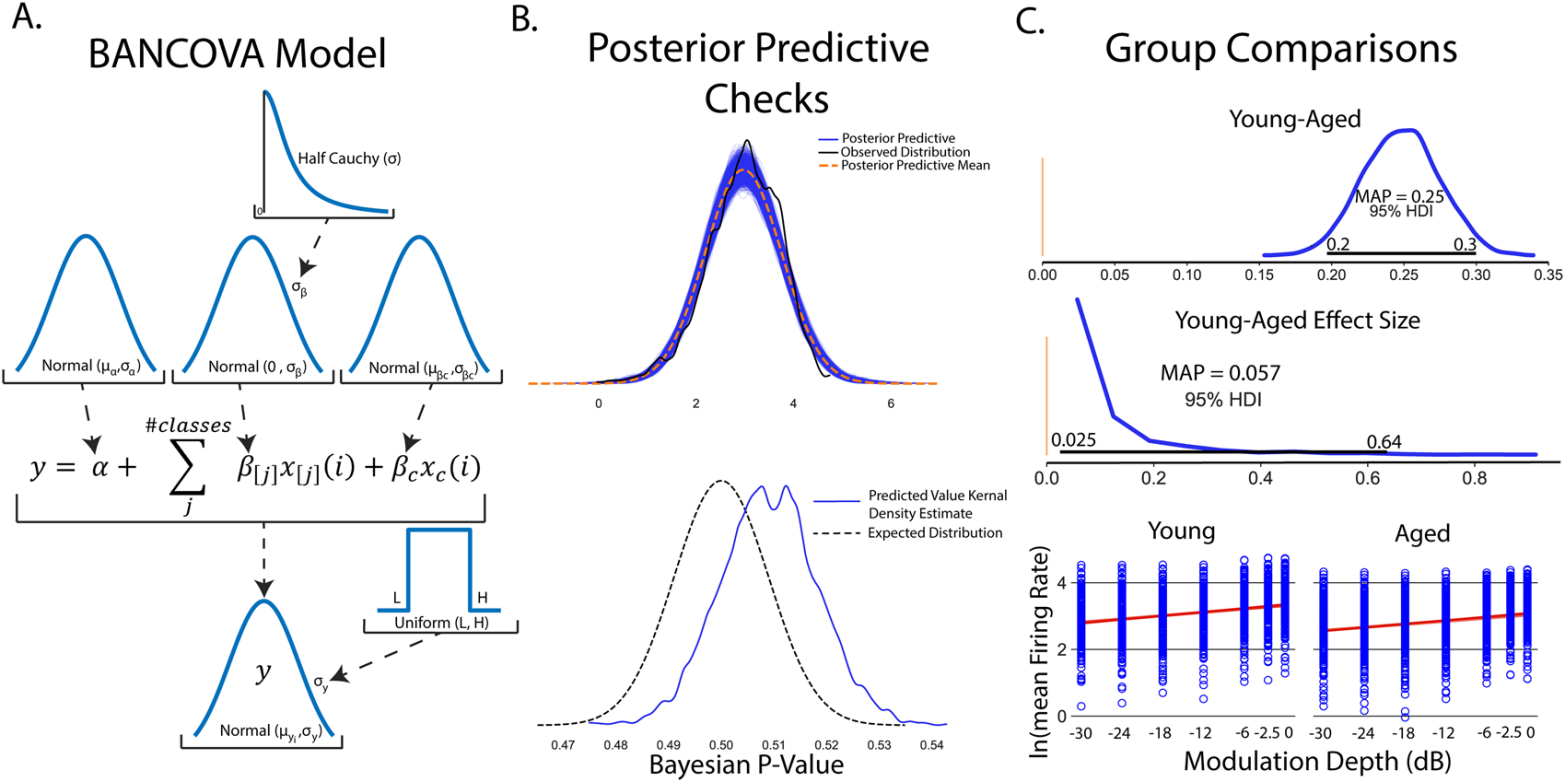
An example of Bayesian inference using ANOVA-like models. ***A***, General schematic of BANOVA/BANCOVA models. Traditional ANOVAs have two key assumptions: normality of group data and homogeneity of variance. Normality of group data is imposed in BANOVA-like models as normal distributions around group parameters with homogeneity of variance encoded as a uniform distribution around posterior variance term $\sigma_{y}$. Traditional ANOVAs assume a fixed variance on group parameter values $\sigma_{\beta }$, imposing the constraint that each group is estimated independently from each other group. A uniquely Bayesian approach is to instead learn $\sigma_{\beta }$ values from the data itself by placing a distribution on $\sigma_{\beta }$. ***B***, Posterior predictive checks suggest that posterior distributions show good fit in mean and variance to observed data. ***C***, Once posterior distributions are calculated, group comparisons can be easily done by subtracting young and aged posteriors to yield a contrast distribution. It is found that firing rates across all modulation depths are significantly higher in aged than young rodents (contrast MAP = 0.25; 95% HDI does not overlap 0). Another unique feature of Bayesian approaches is their ability to assess distributions on the effect size. In this BANCOVA, while group differences are significant, their relative effective size is small but significant (effect size MAP = 0.057; 95% HDI does not cross 0), suggesting marginal impact of age on firing rates elicited from SAM stimuli. Finally, metric covariates of firing rate in response to varying SAM depth in young and aged groups can be plotted as regressions superimposed on raw data.

Bayesian analogs of ANOVA and ANCOVA can be easily defined in PyMC and are termed Bayesian analysis of variance (BANOVA) and Bayesian analysis of covariance (BANCOVA) (Fig. 8*A*), respectively, to distinguish models from their frequentist counterparts. Traditional ANOVAs make two key assumptions; (1) that underlying data is normally distributed and (2) there exists a homogeneity of variance among groups. To account for these assumptions, normal distributions are placed on prior parameter and observed data distributions, and a uniform distribution prior is placed on observed data variance $\sigma_{y}$. Importantly, observed data distributions should be assessed to ensure distributions are normally distributed. While not strictly an ANOVA structure, an advantage of Bayesian approaches is their ability to create models that handle arbitrary distributions. While traditional ANOVAs also assume independent group variances, the relative shared influence between groups can be learned from the data by imposing a hyperprior on group variance $\sigma_{\beta }$ (Gelman, 2006). As with any prior distributions, selection of $\sigma_{\beta }$ should be informed by prior inspection of the data. A half-Cauchy distribution is once again chosen as it is weakly informative and allows for extreme values if data dictate (Gelman, 2006; Polson and Scott, 2012). Setting $\sigma_{\beta }$ to a large constant replicates a traditional ANOVA.

As a guiding example, consider a similar experiment to that done in simple linear regression. In this experiment, we aim to understand age-related changes in the IC auditory processing of SAM sounds. This experiment consisted of two groups of young (animals <6 months in age) and aged (animals >22 months in age). SAM stimuli at increasing modulation depths were played to the animals with evoked single-unit responses recorded from the IC. As seen in the previous simple linear regression experiment (Fig. 4), there is a significant increase in evoked firing rate with increased modulation depth in young animals. As such, it should be included in comparison between the two groups. Taken together, this suggests that BANCOVA will serve as an appropriate model. BANCOVAs are inherently hierarchical (Gelman, 2005; Kruschke, 2014) (Fig. 8*A*) to allow for between-subject variances to be represented in the prior if these variances mutually inform one another. Setting this hyperprior to a constant creates a model analogous to a frequentist ANCOVA (Kruschke, 2014). The formation of the BANCOVA is again relatively straightforward:

Code Example 6: creating a Bayesian ANCOVA

**Figure:**
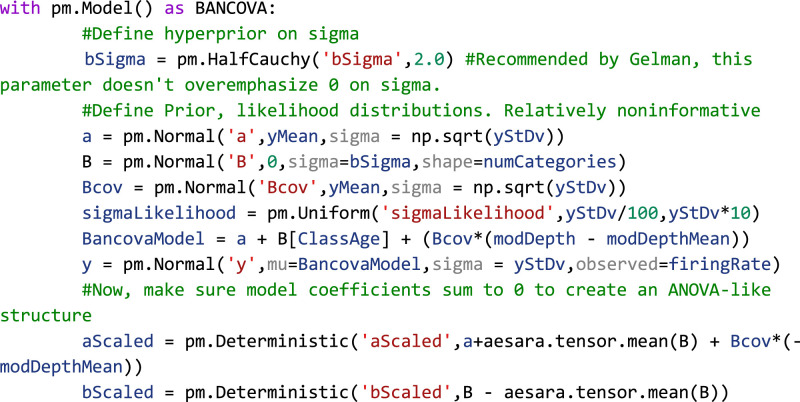

with inference made in the exact same way as the previous models.

After model sampling, posterior sampling checks were performed to ensure posterior distributions adhere well to observed data. Posterior predictive distributions show good qualitative fit to observed firing rate data with Bayesian *p*-values centered ∼0.51, suggesting good model fits to observed data (Fig. 8*B*). Comparisons between groups are simple once posterior distributions are obtained. Similar to Bayesian group comparisons (BEST; Fig. 6), all that needs to be done is to measure differences between aged and young group mean parameter posteriors (Fig. 8*C*), encoding influence of young and age groups on firing rates. Aged and young contrasts show significantly elevated firing rates in young rats across all SAM stimuli (young-aged difference MAP = 0.25; 95% HDI excludes 0). Another advantage of Bayesian inference is the ability to observe the distribution and thus the most likely value and spread of effect size. In this analysis, the effect of age in the SAM stimulus processing is significant but small (effect size MAP = 0.058; 95% HDI excludes 0) but with a wide spread of effect (95% HDI between 0.025 and 0.64), suggesting variable temporal acuity between rodent subjects. Finally, firing rates versus SAM amplitude depth for each class are plotted with $y=\alpha +\beta_{\mathrm{young}/\mathrm{age}}x_{\mathrm{young}/\mathrm{age}}+\beta_{\mathrm{cov}}x_{\mathrm{cov}}$ superimposed.

### Comparisons between frequentist and Bayesian ANCOVAs

To compare BANCOVA results to classical, frequentist ANCOVAs, the young-age IC data underwent *post hoc* ANCOVA analysis in SPSS. Frequentist ANCOVA also found a significant difference between groups after accounting for changes in firing rate from modulation depth (young-age log firing rate = 0.108; *p* < 0.001). The measured confidence interval (there is 95% probability that the 95% confidence interval of 0.087–0.130 calculated from a future sample of data will contain the true parameter value) also suggests some within- and between-group variance. Interestingly, the young-age log firing rate estimates are noticeably smaller in the frequentist ANCOVA than the BANCOVA. The reason for this is due to inclusion of prior knowledge. As described in Figure 8*A*, frequentist ANCOVA and BANCOVA have very similar model structure, with the biggest difference being how the group variance $\left(\sigma_{\beta }\right)$ is defined. Frequentist models impose a point estimate on $\sigma_{\beta }$, while Bayesian interpretations impose a prior distribution. The effect of a point estimate is to pull estimates toward 0 when variances are small (Gelman, 2005) common for ANOVA-like models (Gelman et al., 1996) seen in this data. Bayesian inclusion of a distribution allows for control of this shrinkage by the use of $\sigma_{\beta }$ prior distributions which place higher probability on small variances while also placing small but nonzero probability on larger variances, allowing for high variance data with ample evidence to propagate through the model structure.

### Multiple comparisons in Bayesian inference

In traditional frequentist analyses, corrections for multiple comparisons are necessary in order to ensure that maximum Type I errors (false positives) are constrained to a maximum of 5% (α=0.05). With Bayesian inference, a posterior distribution across all parameters is obtained which remains unchanged no matter how many comparisons are made (Kruschke, 2014). Furthermore, frequentist Type I errors are classically defined in the context of rejection of a null hypothesis. Bayesian inference is not strictly concerned with rejection of a null hypothesis, but is concerned with weighing competing hypotheses given observed data. Bayesian models are not immune to making false conclusions about data, however. These errors, called Type M for errors in magnitude and Type S for errors in sign, occur when outliers in data exert too much influence on inference. These errors can be controlled by proper choice of priors or by building hierarchical models (Figs. 7*A*, 8*A*) which can account for outliers by pulling parameters toward group means when evidence is small and allowing parameters with good evidence to remain. This shrinkage is due to partial pooling, a phenomenon implicit to hierarchical structures which allows for both the quantification of group differences and group similarities(Gelman et al., 2009).

## Discussion

The Bayesian inference approache provides a powerful statistical tool that encourages deep and meaningful exploration of data and allows for presentation of data in intuitive and transparent ways. In this tutorial, we demonstrate the ease by which Bayesian inference can be performed across a wide variety of experimental designs and provide source code that can be modified to accommodate neuroscientific experiments using all freely available and open-source tools. We intentionally used the base PyMC toolchain in order to explicitly show Bayesian model creation. However, there are PyMC plug-in tools such as Bambi (Capretto et al., 2022) which can facilitate creation of Bayesian models in single lines of code. An example of Bambi-enabled model creation is provided in our Bayesian inference toolbox.

### Applications of Bayesian inference

In this tutorial, our inference examples largely focused on data commonly found in electrophysiology and computational neuroscience studies. However, Bayesian inference is agnostic to the form and type of data used in inference. The described statistical models are easily adapted to electroencephalography, neuroanatomical measures, behavioral measures, and calcium events, among others. Bayesian inference is also of particular interest to neuroscience experiments involving very large datasets, such as spatial transcriptomics (Gregory et al., 2022; Song et al., 2022).

We also focused primarily on canonical statistical model structures of *t* test group comparisons, simple regression, ANOVAs, and mixed-effect models. Bayesian models can further be defined for other statistical models such as logistic, Poisson, and ridge regressions, mixture of Gaussians, and time-series analysis.

### Tempering expectations of Bayesian inference

Despite the enthusiasm of some Bayesian advocates, Bayesian inference is not a panacea. It is subject to similar problems as frequentist NSHT, in that models can be used which do not adequately fit underlying data statistics or that priors can be chosen which dominate model performance and de-emphasize observed data. However, Bayesian approaches support and encourage model transparency, requiring researchers to declare model priors and posteriors while encouraging continued discussion of data inference as opposed to stopping if a *p*-value is below an arbitrary threshold. A second caveat is that running MCMCs can be slower than frequentist approaches, with run times occasionally in minutes as opposed to seconds. However, time increases are not astronomical and can be further reduced to levels similar to frequentist approaches by using GPU computing or using programs such as JASP (Love et al., 2019) which utilize a C backend to speed up computation.

### The controversy of the prior

The prior is arguably the most contentious aspect of Bayesian inference, with arguments that the prior unduly influences decisions on data. It is absolutely possible to have priors that distort posterior distributions into poor inference. Similar arguments can be levied at frequentist approaches that perform similar distortions on decision metrics, such as applying ANOVA tests when the underlying data are not normal. Often times, these mistakes are not done out of malevolence, but due to the modern framework of how statistics is performed. We argue that having to consider what prior to use, and thus what one's assumptions are, what distributions are physiologically relevant, and the distributions of observed data will help to prevent errors in statistical modeling while creating greater transparency in how conclusions on data are drawn.

### Decisions with Bayes’ factors

Some studies that utilize Bayesian inference use a decision metric called the Bayes' factor, which is a measurement of the ratio of marginal likelihoods of two competing models providing log likelihood of evidence for one model over another (Johnson et al., 2023). We intentionally chose not to utilize Bayes' factor metrics because, in the authors' opinions, they reduce inference to evaluation of a single metric over an arbitrary threshold, as opposed to analysis over posterior distributions of observed data. Furthermore, certain prior declarations yield undefined Bayes' factors (Gelman and Rubin, 1995) potentially encouraging the use of suboptimum models in order to provide arbitrary decision metrics.

### Bayesian and frequentist approaches: a holistic approach to inference

Following in the steps of Bayarri and Berger (2004), data analysis should not consist solely of Bayesian or frequentist approaches devoid of the other. There are certainly cases where frequentist approaches should be used, such as clinical trials where preregistration and proper protocol design can provide bounds on false-positive and false negative rates necessary for translation of medical therapeutics. Hybrid frequentist and Bayesian approaches can also provide richer insight into analyses where posterior distributions are unidentifiable or difficult to sample (Raue et al., 2013) or in identifying when improper models have been chosen (Berger et al., 1997). Bayesian ideas of posterior predictive checks and model comparisons can also be applied to frequentist NSHT, many of which would help address problems of replication and data transparency. As frequentist approaches are often baked into the pedagogy of neuroscience and neural engineering, we aim for this tutorial to be a thorough introduction into the application of Bayesian statistics to help develop a toolkit that can be used for robust data analysis or in conjunction with previously established frequentist approaches. These models are also easily extendable into Bayesian analogs of logistic or multinomial regressions, Gaussian mixture models, and Bayesian time-series analyses, among many more.

## Code and Data Availability

The code/software, data, and SPSS files described in this article are freely available online at https://github.com/bscoventry/Practical-Bayesian-Inference-in-Neuroscience-Or-How-I-Learned-To-Stop-Worrying-and-Embrace-the-Dist and permanently indexed in a Zenodo repository: 10.5281/zenodo.11206116. The code is available as Extended Data.

## Code and Data Repository

https://github.com/bscoventry/BayesianNeuralAnalysis doi.org/10.5281/zenodo.11206116.

## Synthesis

Reviewing Editor: Arvind Kumar, KTH Royal Institute of Technology Decisions are customarily a result of the Reviewing Editor and the peer reviewers coming together and discussing their recommendations until a consensus is reached. When revisions are invited, a fact-based synthesis statement explaining their decision and outlining what is needed to prepare a revision will be listed below. The following reviewer(s) agreed to reveal their identity: Nicholas Timme, Jingwen Li.

Synthesis

The manuscript presents a tutorial about Bayesian inference in neuroscience. The two reviewers think that this tutorial can serve an important purpose as Bayesian analysis is becoming more popular. But the have raised some concerns about the presentation of the contents and highlight the absence of a comparison with frequency based stat. So we invite you to revise the manuscript according to the reviewers' suggestions. In particular we suggest that you

Describe theory using examples - essentially walk through the theory using data - this is what is expected of a tutorial.

Compare the Bayesian approach with frequency-based approach. This will help reader appreciate the essence of Bayesian method

Personally I would also suggest that you can mention Cinotti and Humphries [https://www.jneurosci.org/content/42/8/1417] which is a nice example of the application of the Bayesian method to pool different experimental data to make the best estimate of network connectivity in the striatum.

Below are the detailed comments from the reviewers.

Reviewer 1

Summary: The authors present an overview of Bayesian analysis for neuroscience data. They include analyses of example data sets to demonstrate how these types of analyses operate.

General Opinion: Overall, I'm excited about this paper and I think it has the potential to be a very helpful resource for the field. I am not an expert in Bayesian analysis, though I am generally familiar with it. Thus, I tried to read the paper from the perspective of the paper's target audience. As such, I believe there are several areas in which the paper could be improved to increase its impact on the field.

Larger Concerns

Lines 71-78: I think it would be helpful to clearly lay out some of the data sets you will be using. The transition to the Materials and Methods section is a little harsh.

I think the Materials and Method section could benefit from some additional subsections (e.g., computational hardware) and some revisions. For instance, the title on line 93 is "results-ish". I would suggest just having methods for each data set with a simple title like "Inferior Colliculus Recordings in ..." or something like that. Lines 161-164 are very important, but I think they would be more appropriate (and helpful to the reader!) in the results and/or discussion. Also, there should be some discussion of your code, your github repository, etc. in this section also, including a step by step guide to running your code or information pointing users to a step by step guide on your github repository.

I understand how you want to start the results with data to drive intuition, but the jump after the first section (starting on line 166) to the details of Bayes' theory and other mathematical details is too big of a leap. You don't return to these data until line 365. I would suggest moving the data until after you have gone through some of the theory details or (probably better) walk through the theory with the data. For instance, can you recast Bayes' rule in terms of the actual data you are talking about? What are E, H and theta in your data? Alternatively, you might consider walking through the mathematical details of Bayesian analysis with a super simple example "experiment" that could even have made up data. What about a hypothetical neuron that encodes luminosity, sound level, or auditory stimulus frequency linearly with firing rate? Having some data to talk through the various terms in Bayes' theory will be very helpful. You can show the reader how a good or bad prior would affect the analysis. You can show the reader how the three different types of inference would really work.

Figure 1 C: I think the legend is incorrect here. It talks about Bayesian model comparisons. Also, how is the kernel density estimate performed?

Lines 312-315: I'm struck by how this section is of extreme practical importance to readers thinking about how to employ Bayesian analyses. Perhaps this information could also be included in the introduction ("Look at these types of inference Bayesian analysis can do! Here are some examples from neuroscience for all these! This rocks!")

Along these same lines, there really isn't much in the introduction in terms of types of neuroscience data that have been used in Bayesian analysis or example citations to neuroscience applications. It would be helpful for neuroscientists who work with all sorts of different types of data to be able to find their type of data at least discussed.

Lines 316-317: Obviously, there are more types of models in neuroscience than models of neural firing rates related to stimuli. This should be rephrased as an example and it might be helpful to include other types of analyses.

Code examples 1 and 2: There isn't much information supplied in terms of how the data are formatted or how to run this code. If you provide more information about these details, it would be helpful to point to this around these sections. I don't necessarily think you need to get bogged down in these details here, but it would be helpful to point the reader to where those details can be found.

Minor Concerns

Line 38, perhaps "the use of p-values to determine significant results"? The use of p-values isn't the decision rule. The decision rule in frequentist approaches uses p-values to determine significant results.

Line 184: What is AM? I don't think you defined this. Is it amplitude modulation? If so, what is amplitude modulation depth? The magnitude of the modulation? (I now see it better explained in the figure, but hopefully my confusion from the text demonstrates that more explanation is needed.)

Line 197: A citation would be helpful here.

Figure 1 A isn't doing much. You can't see how many data points there are for the discrete modulation depths that were tried. What about violin plots or box and whisker plots? I understand you want to plot the data this way because of the model you will apply, but the current format makes it very hard to see individual data points.

Line 236: Should P(H) be P(Theta|M)?

Line 439: Should this reference Figure 4 instead of Figure 3? Also, for other figure references in this paragraph?

Line 456: "analysis our" should probably be "analysis in our".

Reviewer 2

The authors exploit Bayesian inference to analyze and interpret data in neuroscience by first describing and introducing the basics of Bayesian inference and then providing examples of applying Bayesian inference on two datasets. The authors provided the code for implementing the Bayesian inference analyses using an open-source Python package and reported the results on the two example datasets. I found the topic and the analyses interesting and meaningful, as they provide a potential alternative and complement to the traditional frequentist-based testing in neuroscience which is sometimes used without carefully thinking about the underlying assumptions and interpretations. However, the insight and contribution that the manuscript provides are limited and ambiguous with the current analysis and manuscript organization. Below are my concerns and suggestions that I hope could help improve the manuscript.

Major concerns:

1) The manuscript does not provide any comparison between the frequentist-based testing and Bayesian inference in analyzing the two example datasets. Are the results from the two different approaches consistent with each other or do they differ? What new insight does Bayesian inference bring that frequentist-based testing does not? It is crucial to discuss the consistency and difference between the two approaches, the advantages and disadvantages of each approach, and the benefits and advances in using Bayesian inference.

2) The main result of the manuscript comes from applying the Bayesian-based analyses on the two example datasets. Neither the Bayesian inference methods nor the computational toolbox is original and novel in this manuscript. Thus, the description of Bayesian inference and the example code of using the computational toolbox should be put in methods instead of taking the extensive space in the result section.

3) Relatively, I did not find any code/software provided as Extended Data in addition to the code provided in the main text. In addition to the example code, the authors should provide well documented code/software, and make sure it is applicable to other datasets as the goal of the manuscript is to provide a paradigm of Bayesian inference in neuroscience.

Minor suggestions:

1) In the abstract, it is a little misleading that the authors wrote "using data from a variety of neuroscientific studies" and "providing an open-source toolbox". From what I read in the paper, the authors used two example datasets in neuroscientific studies and provided example code to use an existing open-source toolbox. I suggest the authors rephrase these claims in the abstract to better clarify the scope of the manuscript.

2) Are the two datasets used in the manuscript published from other papers, or unpublished from the authors' own experiments? If they are published data from other papers, I suggest the authors explicitly cite the papers in line 101 and line 131 when mentioning "this dataset" and "in this study". I am not sure if the datasets are data from all the papers cited in lines 83-86. In addition, the citations "Palombi et al., 2001; Simon et al., 2004; C.F et al., 2012" in lines 83-84 seem missing in the references.

3) In line 53, "the problem of the p-value" may misleadingly suggest an inherent problem with using p-value in neuroscience. However, it is how people use and interpret p-values where problems may exist.

Typos:

Fig. 1, there are two "C" in figure caption, the second "C" doesn't seem to have corresponding panel in the figure and is not used in the main text neither.

Line 184, "AM" should be "SAM"?

Line 368, "data observed data"

Line 399, "Fig. 3a" should be "Fig. 3A"

Line 431, here should cite Fig. 3C?

Line 441 and 444, "Fig. 3A" and "Fig. 3B" should be "Fig. 4A" and "Fig. 4B"

Line 575, "Fig. 5A" should be "Fig. 6A"

Line 591, "Fig. 2" should be ?

## Author Response

Dear Dr. Kumar, Thank you for the opportunity to resubmit a revised version of our manuscript "Practical Bayesian Inference in Neuroscience: Or How I Learned to Not Worry and Trust the Distribution" for publication in eNeuro. We appreciate the continued time and effort that you and the reviewers have taken to provide feedback and valuable improvements to our paper. We have incorporated the suggested changes into our paper. These changes are highlighted in blue bold-faced text. Please find below a point-by-point response to reviewer comments and concerns with page numbers referenced to the revised manuscript.

Synthesis of Reviews:

Computational Neuroscience Model Code Accessibility Comments for Author (Required):

Code is available in extended data and as a freely available and archived GitHub Repository: https://github.com/bscoventry/Practical-Bayesian-Inference-in-Neuroscience-Or-How-I-Learned-To-Stop-Worrying-and-Embrace-the-Dist and our permanent Zenodo repository: 10.5281/zenodo.11206116. Code base has been updated to include additional code used in addressing reviewer's concerns.

Synthesis Statement for Author (Required):

Synthesis The revised manuscript has addressed much of the reviewer's concerns but there are a couple of issues that require some more edits in the manuscript.

Reviewer #1 Overall, I found the paper to be much improved from the previous version. I appreciate the thoughtful responses and updates the authors provided. However, I believe additional edits will strengthen the paper.

Thank you very much! Additional edits are addressed below.

I would suggest walking through the theory (lines 106-275) with a simple example. As it is, I think it is a good walkthrough of Bayesian analyses, but having a simple model along the way will be helpful. What I suggest is basically keeping all the material in this section and adding additional material along the way that connects to an example. I think you could take the material in your hypothetical in vitro ephys example (lines 277-317) and use that to help the reader understand the terms in Bayes rule. For instance, on line 137, I would suggest a new paragraph along these lines: "This can be really confusing without concrete examples, so let's consider example experiment and analysis X. (A few details on X.) In this hypothetical analysis M is ..., theta is ..., E is .... We'll walk through each of the terms in Bayes' rule and connect them to this hypothetical analysis." Then, in each of the subsequent sections, you add material connecting the various terms to the hypothetical example.

Thank you for your suggestion. We agree that concrete examples of prior and posterior distributions and likelihood functions along with theory would aid the reader in the understanding and employ of Bayesian methods. We have adapted our Bayesian example to be incorporated into Bayesian Theory.

We begin by introducing our motivating hypothetical experiment in lines 104-116. We then move to the theory of Bayes' rule, updating the example during discussion of the prior (lines 196-201), likelihood function (lines 228-239) and posterior distribution and inference (lines 300-323).

You don't really compare how a frequentist approach would analyze the IC data. I think this comparison would be helpful. Along these lines, I think more direct comparisons to frequentist approaches (where you actually apply the frequentist approach, not just talk about its problems) would be helpful throughout the paper. I know the editor and the other reviewer emphasized this point in particular and I agree with them.

Thank you very much for the suggestion. We have added frequentist analyses for the IC regression (752-802) and IC young-aged ANCOVA models (1016-1033) that will complement the comparison frequentist t-tests performed in the Bayesian comparison of groups models (819-856, Figure 6D). Frequentist analyses were performed using the validated and established SPSS software, with SPSS code added to our code repository. The example of frequentist vs Bayesian regressions show cases where the two methods come to the same conclusion. However, frequentist approaches offer a paucity of information compared to Bayesian inference. The ANCOVA vs BANCOVA example shows how the two inference paradigms can diverge and discusses how ANOVA-like models can bias differences in groups in experiments with smaller variances.

However, we believe it is important to state that post hoc frequentist analyses as performed in this paper do not follow best inference design for frequentist inference. We recognize that proper frequentist design is not always followed in the analysis of data, and note this in the manuscript (lines 368-370, 799-802). To address this, we added a section detailing how unseen bias can be introduced into frequentist inference due to ill-defined stopping rules (lines 390-453, Fig 2D-E) to complement our section on performing frequentist inference (lines 364-388). While our focus in this article is on performing Bayesian inference, we do believe each statistical paradigm has its proper application and use. We hope that discussion of the proper frequentist inference design (lines 364-388) and what can go wrong when not followed properly (lines 390-453) can serve as a good pedagogical example and can help with more robust and reproducible frequentist inference.

Is figure 2 B just a specific modulation depth from 2A? I don't understand the relationship between Figure 2 A and B. Please clarify.

Thank you for identifying this section. As per reviewer 2 comments, a portion of figure 1 was split into a separate figure, making figure 2 referenced here figure 3 in the revised manuscript.

Figure 3A is meant to show the distribution of responses as a function of modulation depth. Figure 3B is the distribution of firing rates across all modulation depths for the full study. Observation of the entire firing rate distribution is helpful in determining a likelihood function for Bayesian inference. We have adjusted inline text (lines 625-627,638-643) and figure legends to better describe panels A and B (lines 1409-1416).

Line 65 seems to indicate that Bayes' rule was developed after the frequentist approaches, but line 228 indicates the opposite.

Thank you for this suggestion! Our intention was to state that Bayesian approaches were developed earlier than frequentist methods, but Bayesian inference was constrained to certain canonical forms, leading to the adoption of frequentism when it was developed. We have clarified the text (lines 63-66).

For the section that starts on line 642, I'm confused about what Bayesian model you constructed to perform this analysis. In the previous example, it was a linear model and I understood what the parameters were for which the Bayesian methods produced posterior distributions. I don't see how Bayesian methods are being applied in this example.

The model described in this section is the Bayesian Estimation Supersedes the T-Test (BEST), a well-established and validated Bayesian model for the comparison of groups, serving as the Bayesian analog of the t-test. We have added text in the previous section describing t-test statistical models to make this clearer (lines 815-817).

Line 229 has some grammar issues.

Thank you for pointing out these grammar issues. We have rewritten this section of the article to be more clear and concise (lines: 249-262).

Line 1182, I don't see a biorender image in the panel. Perhaps I'm missing something? Thank you for pointing this out! BioRender was used in the figure for the initial design of 1E, but was removed later. We have removed this reference to Biorender.

I think something went wrong in the formatting of Table 1, rows 3 and 4.

Thank you for pointing this out. This was a formatting issue found in the manuscript response but not the clean manuscript. This has been corrected (lines 1560).

Lines 620-625 should probably be in the methods or at the end of the Bayes rule theory section, not the middle of the results.

Thank you, we agree. This has been moved to the end of the methods section (line 601-606).

Do you define what DBS stands for? If you did, I missed it.

Thank you for identifying this. It was previously described in the materials and methods section, but on subsequent review, we realized that the description of DBS would benefit from a more detailed description. This has been adjusted in lines 579-584.

The semicolon on line 696 should probably be a hyphen. "a so-called dose-response curve" is not a complete sentence.

Thank you, this has been corrected in the manuscript (line 870).

Reviewer #2 The structure of the paper has been incredibly improved in the revision. I agree with the authors to move the Bayesian inference theory out of results as an introduction, and thus the results focus on going through the application of Bayesian inference analyses on two examples. It largely helps the readers to follow and learn. The more specific subtitles also helped improve the clarity of the structure.

The comparison in Fig. 5D strongly illustrates one of the advantages of Bayesian inference over frequentist p-values.

The authors well answered and addressed my concerns. I appreciate the authors' effort in improving the manuscript. I will be glad to see the manuscript being accepted.

Thank you very much! We have also addressed the minor suggestion below.

Here is one minor suggestion the authors could consider to further polish the manuscript:

Please better explain the meaning of each panel in Fig 1E in the figure caption. I could easily get it since I have read through the manuscript. However, as the first figure, a more detailed explanation here might be helpful for the first-time readers.

Thank you, we agree this could be made clearer. We have split out Figure 1E into a part of a new figure 2. We have panelized each section of previous 1E into panels 2A, 2B, and 2C showing observed data distributions in A, potential prior choices in 2B, and inference using each prior in panel C. We have also adjusted the text (lines 105-107,198,228-239,300-323) and figure legends (lines 1397-1408) to provide more detailed descriptions of the figure.

We sincerely thank the editor and reviewers for their helpful feedback and constructive criticism.
